# Disruption of memory B-cell trafficking by belimumab in patients with systemic lupus erythematosus

**DOI:** 10.1093/rheumatology/keae286

**Published:** 2024-05-22

**Authors:** Eline J Arends, Mihaela Zlei, Christopher M Tipton, Jasna Cotic, Zgjim Osmani, Fenna J de Bie, Sylvia W A Kamerling, Andre van Maurik, Richard Dimelow, Yun Irene Gregan, Norma Lynn Fox, Ton J Rabelink, David A Roth, Ignacio Sanz, Jacques J M van Dongen, Cees van Kooten, Y K Onno Teng

**Affiliations:** Expert Center for Lupus-, Vasculitis-, and Complement-Mediated Systemic diseases (LuVaCs), Department of Internal Medicine—Section Nephrology, Leiden University Medical Centre, Leiden, The Netherlands; Department of Immunology, Leiden University Medical Centre, Leiden, The Netherlands; Medical Laboratory, Department of Flow Cytometry, Regional Institute of Oncology, Iasi, Romania; Lowance Centre for Human Immunology, Emory University School of Medicine, Atlanta, GA, USA; Department of Medicine, Division of Rheumatology, Emory University, Atlanta, GA, USA; Clinical Statistics, GSK, Middlesex, UK; Expert Center for Lupus-, Vasculitis-, and Complement-Mediated Systemic diseases (LuVaCs), Department of Internal Medicine—Section Nephrology, Leiden University Medical Centre, Leiden, The Netherlands; Department of Immunology, Leiden University Medical Centre, Leiden, The Netherlands; Expert Center for Lupus-, Vasculitis-, and Complement-Mediated Systemic diseases (LuVaCs), Department of Internal Medicine—Section Nephrology, Leiden University Medical Centre, Leiden, The Netherlands; Clinical Pharmacology and Experimental Medicine, GSK, Hertfordshire, UK; Clinical Pharmacology Modelling and Simulation, GSK, Hertfordshire, UK; Clinical Science Immunology, GSK, Collegeville, PA, USA; Clinical Development, GSK, Collegeville, PA, USA; Expert Center for Lupus-, Vasculitis-, and Complement-Mediated Systemic diseases (LuVaCs), Department of Internal Medicine—Section Nephrology, Leiden University Medical Centre, Leiden, The Netherlands; Research and Development, GSK, Collegeville, PA, USA; Lowance Centre for Human Immunology, Emory University School of Medicine, Atlanta, GA, USA; Centro de Investigación del Cáncer-Instituto de Biología Molecular y Celular del Cáncer (CIC-IBMCC, USAL-CSIC-FICUS) and Department of Medicine, University of Salamanca, Salamanca, Spain; Expert Center for Lupus-, Vasculitis-, and Complement-Mediated Systemic diseases (LuVaCs), Department of Internal Medicine—Section Nephrology, Leiden University Medical Centre, Leiden, The Netherlands; Expert Center for Lupus-, Vasculitis-, and Complement-Mediated Systemic diseases (LuVaCs), Department of Internal Medicine—Section Nephrology, Leiden University Medical Centre, Leiden, The Netherlands

**Keywords:** biologicals, B-lymphocyte, gene expression, SLE, LN

## Abstract

**Objectives:**

Autoreactive memory B cells (MBCs) contribute to chronic and progressive courses in autoimmune diseases like SLE. The efficacy of belimumab (BEL), the first approved biologic treatment for SLE and LN, is generally attributed to depletion of activated naïve B cells and inhibition of B-cell activation. BEL’s effect on MBCs is currently unexplained. We performed an in-depth cellular and transcriptomic analysis of BEL’s impact on the blood MBC compartment in patients with SLE.

**Methods:**

A retrospective meta-analysis was conducted, pooling flow cytometry data from four randomized trials involving 1245 patients with SLE treated with intravenous BEL or placebo. Then, extensive MBC phenotyping was performed using high-sensitivity flow cytometry in patients with mild/moderate SLE and severe SLE/LN treated with subcutaneous BEL. Finally, transcriptomic characterization of surging MBCs was performed by single-cell RNA sequencing.

**Results:**

In BEL-treated patients, a significant increase in circulating MBCs, in a broad range of MBC subsets, was established at week 2, gradually returning to baseline by week 52. The increase was most prominent in patients with higher SLE disease activity, serologically active patients and patients aged ≤18 years. MBCs had a non-proliferating phenotype with a prominent decrease in activation status and downregulation of numerous migration genes.

**Conclusion:**

Upon BEL initiation, an increase of MBCs was firmly established. In the small cohort investigated, circulating MBCs were de-activated, non-proliferative and demonstrated characteristics of disrupted lymphocyte trafficking, expanding on our understanding of the therapeutic mechanism of B-cell-activating factor inhibition by BEL.

**Trial registration:**

ClinicalTrials.gov, http://clinicaltrials.gov, NCT00071487, NCT00410384, NCT01632241, NCT01649765, NCT03312907, NCT03747159.

## Graphical Abstract

**Figure:**
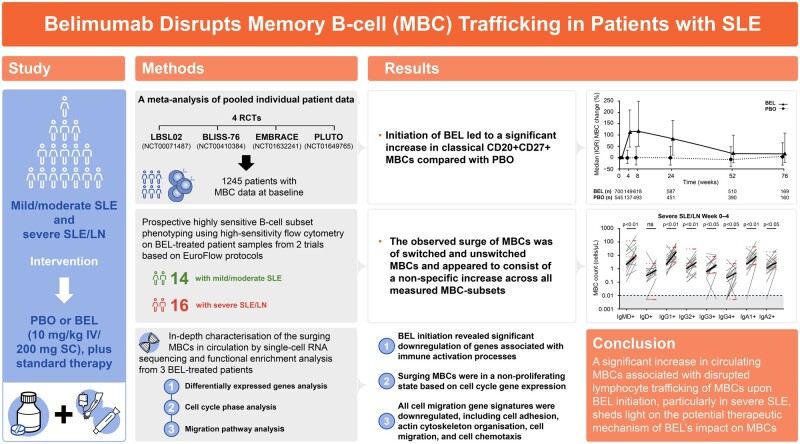

Rheumatology key messages

- In patients with systemic lupus erythematosus (SLE), belimumab substantially increased circulating memory B cells (MBCs).
- Most prominent increase was in patients with high SLE activity, serologically active or ≤18 years old.
- Increases in circulating MBCs potentially allow for a more efficient targeting of the B-cell compartment.

## Introduction

 SLE is an autoimmune disease characterized by autoreactive memory B cells (MBCs) production [1]. Belimumab (BEL), an approved biologic agent for active SLE and LN, reduces circulating B cells through neutralization of B-cell-activating factor (BAFF) [2]. BAFF inhibition by BEL prevents the inhibition of B-cell apoptosis and the promotion of B-cell differentiation into Ig-producing plasma cells [2], resulting in decreases in immature and naïve B cells, while sparing MBCs [3]. Autoreactive MBCs, which have a decreased threshold for activation, are likely to be the major contributors to the observed chronic and progressive course in autoimmune diseases like SLE. Indeed, murine studies have reported that MBC survival is independent of BAFF, suggesting secondary humoral immune responses remain intact despite the absence of BAFF [4]. This is further corroborated by the persistence of adequate vaccination responses in BEL-treated patients with SLE [5]. Previous BEL studies indicated that MBCs might not only be spared but increase in circulation [6, 7], suggesting reduced MBCs in the tissue. This potentially means that MBCs can no longer effectively interact with other immune cells, such as T cells, in well-organized lymphoid structures in the tissues, likely compromising the immune responses. Altogether, little is currently known about the effect of BAFF inhibition on MBCs.

Furthermore, high numbers of MBCs were previously shown to be associated with poor clinical responses to rituximab (RTX) treatment in several autoimmune diseases [8, 9]. Importantly, in patients with SLE and LN, RTX failed to demonstrate superior efficacy to placebo (PBO) in two randomized controlled trials [10, 11].

Data on BEL’s effect on MBC populations are important as circulating autoantibodies persist despite conventional immunosuppressive treatment, even when BEL is employed as an add-on therapy in SLE [12]. This persistence can be explained by either the presence of long-lived autoreactive plasma cells resistant to immunosuppression and BEL, or by residual MBCs capable of mounting an autoimmune response [13]. BAFF mediates sites of survival niches for these long-lived cells [14, 15], and BEL could potentially interfere with this notorious mechanism of therapy resistance, making MBCs susceptible to therapeutic targeting.

This study investigated the hypothesis that BEL initiation in patients with SLE increases circulating MBCs and set out to determine the underpinning mechanism. To do so, a meta-analysis of flow cytometric data was performed in a large cohort of patients with SLE treated with BEL *vs* PBO. Additionally, an in-depth characterization of the longitudinal dynamics of human MBC response post-BEL initiation was performed by employing EuroFlow-based high-sensitivity flow cytometry (HSFC) and single-cell ribonucleic acid sequencing (scRNA-seq).

## Materials and methods

### Study design

A meta-analysis of individual patient data pooled from 1313 patients with SLE treated with intravenous BEL 10 mg/kg or PBO from four randomized, double-blind, PBO-controlled studies (LBSL02 [NCT00071487] [6], BLISS-76 [NCT00410384] [3], EMBRACE [NCT01632241] [16] and PLUTO [NCT01649765] [17]) was performed. Patients without a baseline MBC value were excluded, resulting in 1245 patients with SLE (BEL, *n* = 700; PBO, *n* = 545). Additionally, extensive B-cell subset phenotyping was performed prospectively using HSFC on samples of 14 patients with mild/moderate SLE (SLE Disease Activity Index 2000 [SLEDAI-2K] score ≥6) from the BLISS-BELIEVE study (NCT03312907) [18] and 16 patients with severe SLE/LN (SLEDAI-2K score ≥12) from the SynBioSe-2 trial (NCT03747159) [19]. These 30 patients were ≥18 years of age and treated with subcutaneous BEL 200 mg/week. HSFC for both trials was performed in the same laboratory using the same machinery and analysis techniques. In-depth characterization of surging MBCs in circulation was performed by scRNA-seq in longitudinal paired samples from three patients from the SynBioSe-1 trial before and after BEL initiation [20]. Further detail on B-cell subset HSFC, isolation of classical MBCs by fluorescence-activated cell-sorting, scRNA-seq and statistics are presented in the Supplementary Methods.

Specific details relating to the individual clinical trials utilized for this analysis can be found in their primary publications [3, 6, 16–19].

#### Ethics

This meta-analysis was exempt from ethics approval as data were obtained from previous studies for which informed consent and all necessary approvals had already been obtained for each study by the trial investigators. Therefore, the use of these data for the current analysis adheres to the ethical standards established in original studies.

### Retrospective meta-analysis of flow cytometry data

Flow cytometry data on the absolute peripheral MBC subset (CD19+CD20+CD27+) were previously determined in four clinical trials (LBSL02, BLISS-76, EMBRACE and PLUTO) using a central laboratory. These trials were conducted over a period of 15 years; thus, the gating strategy was not identical across trials. For LBSL02 and BLISS-76, the enumeration of MBCs was derived from back-calculations using the white blood cell count and lymphocyte count from the Gen-S Haematology Analyzer, whereas for EMBRACE and PLUTO, the absolute counts of MBCs were derived from back-calculations using a standard bead-based lymphocyte subset flow cytometry panel. Data from baseline, weeks 8, 24 and 52 were combined from all four studies, week 4 data from studies LBSL02 and PLUTO [6, 17] were combined, and week 76 data were derived from BLISS-76 [3] only. Exclusively on-treatment data from BEL- or PBO-treated patients were considered. Percentage changes from baseline were summarized by visit and summaries of absolute cell counts were added in a *post hoc* analysis. MBC variability was investigated by covariate analysis of week 8 percentage change data (Supplementary Methods).

## Results

### Initiation of BEL treatment causes a surge in circulating MBCs

**Figure 1.**
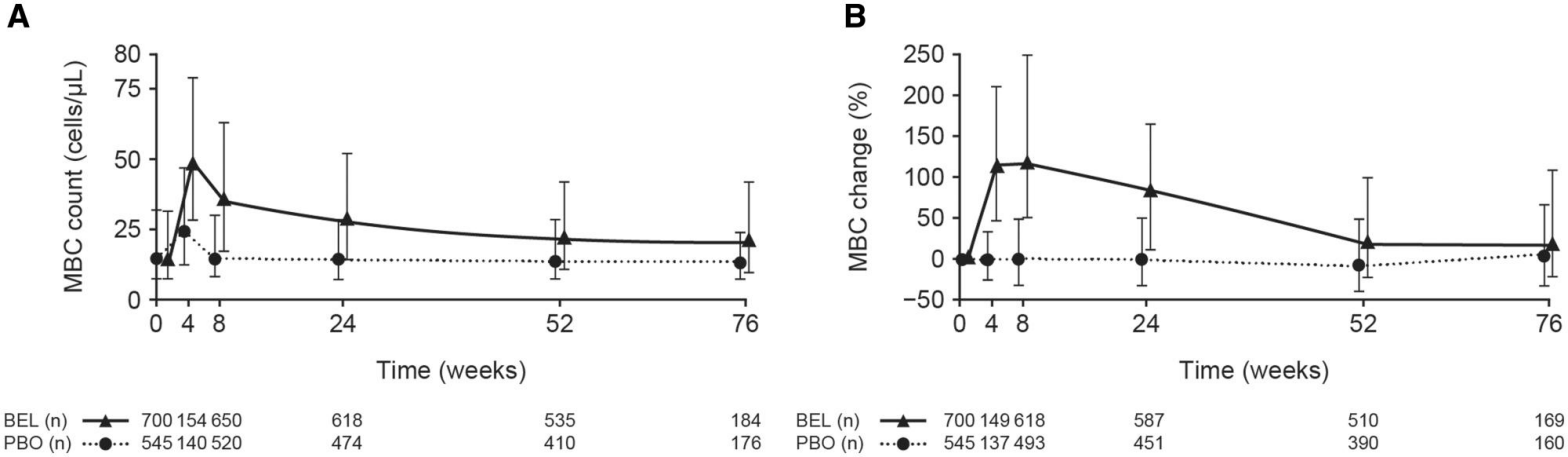
Median (IQR) MBC changes in patients from four pooled SLE clinical trials treated with BEL 10 mg/kg IV or PBO: absolute cell count (*post hoc* analysis) (A), and percentage change from baseline (B). Week 4 data are from studies LBSL02 [6] and PLUTO [17] only, and Week 76 data are from BLISS-76 [3] only. The apparent increase in absolute cell counts at week 4 in the PBO arm is an artefact attributable to minor variations in baseline cell counts in the included studies. Patients with a baseline MBC count of zero were excluded from percentage change calculations in post-baseline visits (B) because of division with zero. BEL: belimumab; IQR: interquartile range; IV: intravenous; MBC: memory B-cell; PBO: placebo

Pooled flow cytometry data showed a considerable MBC increase in BEL-treated patients *vs* PBO treatment at week 4 and week 8, the earliest time points for each study (Fig. 1A). Corresponding increases in median percentage change continued until week 24 in BEL-treated patients (Fig. 1B). By week 52, circulating MBCs had gradually returned towards baseline levels. MBC levels in PBO-treated patients stayed stable over time (Fig. 1). No clear associations for the surge of MBCs were identified (Supplementary Fig. S1, available at *Rheumatology* online). Although significant, a weak correlation without clinical relevance was found between MBC percentage change and BAFF or anti-dsDNA autoantibody levels.

More evident insights into MBC variability were obtained from the categorized covariates (Supplementary Table S1, available at *Rheumatology* online), where the surge of MBCs (median percentage change from baseline to week 8) in BEL-treated patients exhibited consistent, statistically significant increases in subgroups of patients with higher disease activity (SLEDAI score ≥10, anti-dsDNA ≥30 IU/mL and low complement levels). There was a trend for greater percentage increases in MBCs among BEL-treated patients who received steroid or antimalarial concomitant medication at baseline *vs* those who did not. In PBO-treated patients, no statistically significant differences in MBC percentage change associated with disease activity or baseline concomitant medications were found. Of note, the youngest age group (≤18 years) had a statistically significantly larger percentage increase in MBCs *vs* the reference group (19–45 years). This effect was discernible in both the treatment groups but was more pronounced in BEL *vs* PBO.

### Increase in MBCs is not specific for certain Ig subsets

**Figure 2.**
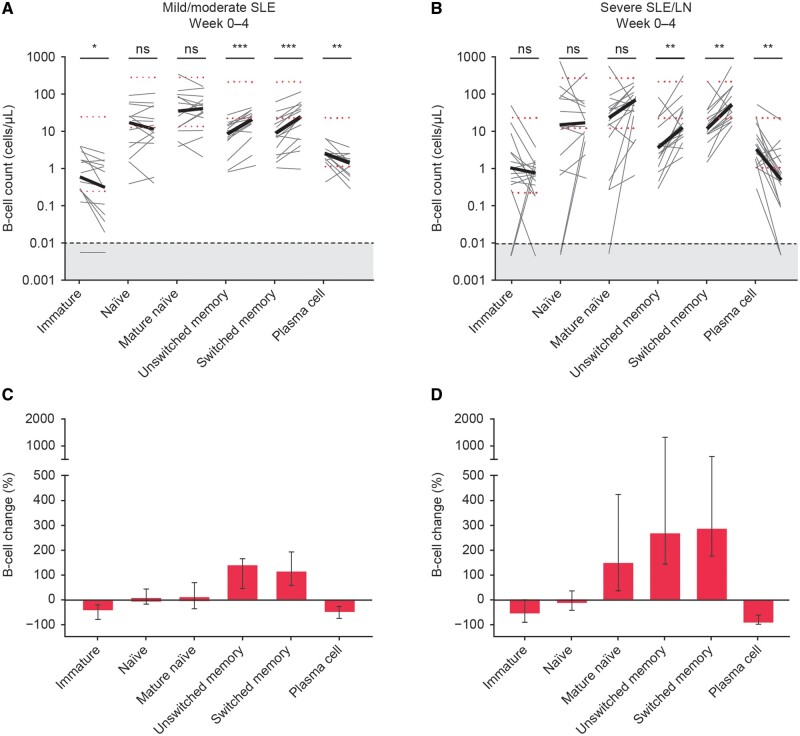
B-cell changes after initiation of BEL measured by high-sensitivity FACS by EuroFlow protocol on samples from 14 patients with mild/moderate SLE (A, C) and 16 patients with severe SLE/LN (B, D). Depicted are changes in B-cell subsets at baseline compared with week 4 in absolute cell counts with the median in bold (A, B) and in median (IQR) percentage change (C, D). The grey area indicates 1–20 analyzed events that were excluded from the analysis. Black dotted line at 0.01 cells/µL indicates the detection limit. Red dotted lines indicate the proposed normal values for adults 18–59 years of age [21]. *P < 0.05; **P < 0.01; ***P < 0.001. BEL: belimumab; FACS: fluorescence-activated cell-sorting; IQR: interquartile range; ns: not significant

HSFC was employed for a more in-depth cellular phenotyping of the surge in MBCs, other B-cell subsets and Ig subsets. Absolute cell numbers and fold changes for different B-cell subsets showed significant increases in circulating unswitched and switched MBCs in both mild/moderate and severe SLE/LN patient groups (Fig. 2). Comparing the fold changes of patients with mild/moderate SLE (Fig. 2A and C) with patients with severe SLE/LN (Fig. 2B and D), a more profound increase was observed in the severe SLE/LN group for both unswitched and switched MBCs. For other B-cell subsets, plasma cells decreased significantly in both the groups. Concurrently, immature B cells in the mild/moderate SLE group decreased significantly. Interestingly, a non-significant increase in mature naïve B cells was observed in the severe SLE/LN group.

**Figure 3.**
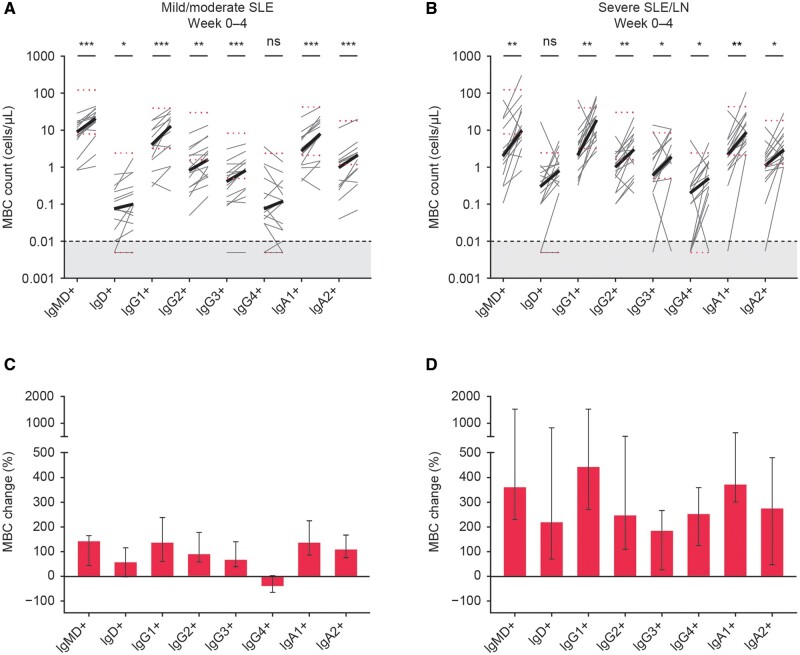
MBC changes after initiation of BEL measured by HSFC by EuroFlow protocol on samples from two different clinical trials including 14 patients with mild/moderate SLE (A, C) *vs* 16 patients with severe SLE/LN (B, D). Depicted are changes in MBCs subclassified according to their Ig isotypes at baseline compared with week 4 in absolute cell counts with the median in bold (A, B) and in median (IQR) percentage change (C, D). The grey area indicates 1–20 analyzed events that were excluded from the analysis. Black dotted line at 0.01 cells/µL indicates the detection limit. Red dotted lines indicate the proposed normal values for adults 18–59 years of age [21]. *P < 0.05; **P < 0.01; ***P < 0.001. BEL: belimumab; HSFC: high-sensitivity flow cytometry; MBC: memory B-cell; ns: not significant

The surge in circulating MBCs was not limited to a particular MBC subset, as significant increases were observed in all assessed MBC subsets in both the patient groups (Fig. 3) except for IgG4+ (Fig. 3A and C) and IgD+ (Fig. 3B and D). These two subsets showed a consistent trend towards a strong increase but were significant in only one patient group (mild/moderate SLE for IgG4+; severe SLE/LN for IgD+), owing to very low starting numbers with a normal range reaching below the detection limit (0.01 cells/µL).

To better understand the kinetics of the MBC surge post-BEL initiation, a subset of nine patients with severe SLE/LN was assessed. In this subset of patients with severe SLE/LN, the MBC increase was already present at week 2, with similar B-cell subset kinetics showing apparent increases in MBCs and all assessed subsets, except for plasma cells (Supplementary Fig. S2, available at *Rheumatology* online).

Notably, we were able to confirm a high level of similarity in MBCs before and after BEL treatment by assessing the sequence density and mutation frequency of the different Ig subsets with scRNA-seq (Supplementary Fig. S3A, available at *Rheumatology* online). As expected, the greatest portion of MBCs comprised IgM, IgG1 and IgA1, and a non-specific increase in all Ig subsets was observed (Supplementary Fig. S3B, available at *Rheumatology* online).

### A decrease in activation status of MBCs is associated with downregulation of migratory processes

The MBC surge may be due to proliferation of MBCs, migration of different MBCs into the circulation or accumulation of MBCs in circulation by reduced extravasation of circulating MBCs. To investigate these hypotheses, we performed scRNA-seq on sorted MBCs before and after BEL initiation using samples from baseline and week 2, as this time point showed the most profound increase in MBC levels in severe SLE/LN.

**Figure 4.**
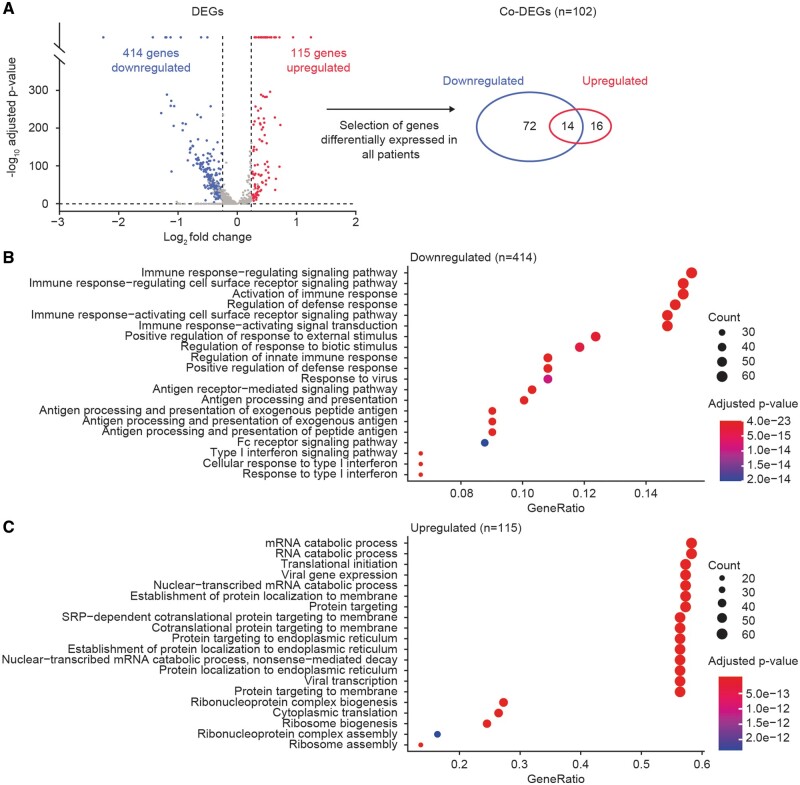
Identification of DEGs and co-DEGs at 2 weeks after BEL initiation. Depicted are the average log_2_ fold changes from genes with a minimal percentage expression of 10% and a change above 0.25 in all analyzed patients. Downregulation is shown in blue, upregulation in red (A), with the corresponding top 20 significant biological processes assessed from functional enrichment analysis on 414 downregulated DEGs (B) and 115 upregulated DEGs (C). BEL: belimumab; DEG: differentially expressed gene; mRNA, messenger RNA; RNA: ribonucleic acid; SRP: signal-recognition particle

The scRNA-seq analysis revealed 529 significant differentially expressed genes (DEG), of which 414 were predominantly downregulated and 115 were upregulated (Fig. 4A). These DEGs are associated with 685 downregulated and 46 upregulated biological processes, respectively. The downregulated processes are all characterized by decreased activation of the immune response *via* a decrease in antigen processing and presentation, a decrease in immune response signalling and a decrease in type I interferon signature (Fig. 4B). The upregulated processes are less specific and can be summarized by an increase in basal cellular processes represented by proteins targeting the endoplasmic reticulum and membrane, ribosome assembly with cytoplasmic translation and messenger RNA degradation (Fig. 4C). Taken together, these findings indicate a decrease in activation status of the MBCs 2 weeks post-BEL initiation.

**Figure 5.**
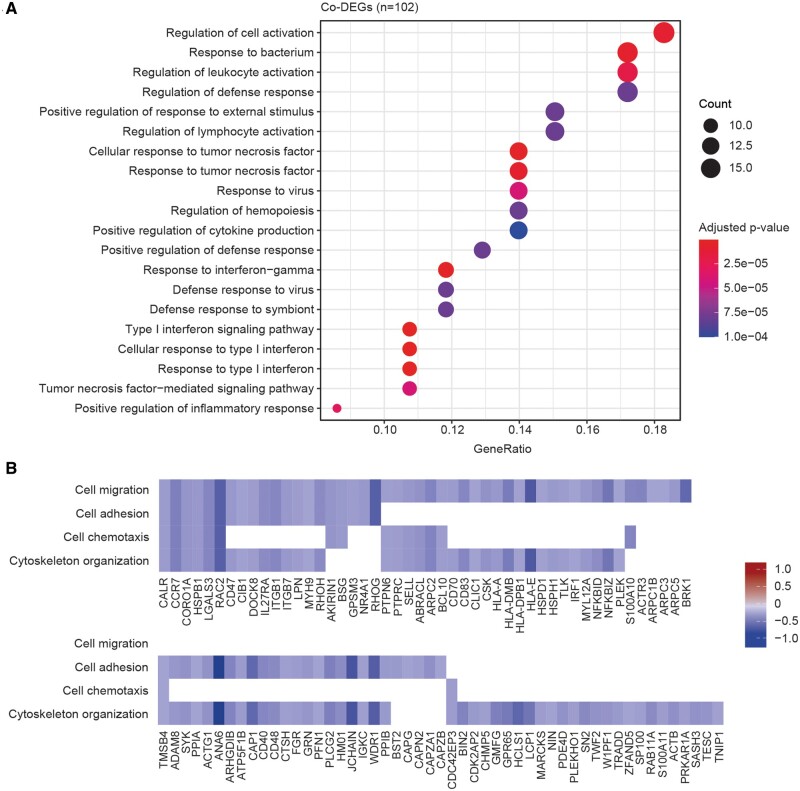
In-depth analysis on DEGs and co-DEGs using FEA on GO terms and migration analysis at 2 weeks after BEL initiation. The top 20 significant biological processes associated with the 102 co-DEGs are shown (A). The average log_2_ fold changes at 2 weeks after BEL initiation of 99 DEGs associated with migration processes are depicted, subdivided into four categories: cell migration, cell adhesion, cell chemotaxis, and actin cytoskeleton organization (B). BEL: belimumab; DEG: differentially expressed gene; FEA: functional enrichment analysis; GO: gene ontology

From the 529 DEGs, 102 co-DEGs were differentially expressed in all patients: 16 upregulated, 72 downregulated and 14 genes with an inconsistent fold change (Fig. 4A and Supplementary Fig. S4, available at *Rheumatology* online). Within the downregulated genes, CD40, CCR7 and SELL (CD62L) are well-known genes associated with cell interactions. Functional enrichment was employed to assess all genes associated with biological processes, yielding results similar to those of the total DEG set (Fig. 5A). Consistent with the treatment, the downstream pathway of BAFF-R, which is the TNF ligand superfamily member 13B that signals through the TNF-mediated signalling pathway (Gene Ontology, GO: 0033209) and Nuclear Factor Kappa B Subunit 2 (NF-κB-2)-inducing kinase (NIK)/NF-κB-2 signalling (GO: 0038061), was significantly downregulated in all patients. In total, 17 GO terms associated with downstream BAFF signalling were significantly downregulated. While BAFF-R itself was not downregulated on the RNA level, the transmembrane activator calcium modulator and cyclophilin ligand interactor receptor (TNFRS13B), which is another survival receptor and a ligand for a proliferation-inducing ligand, and BAFF, to a smaller extent, did have decreased RNA expression.

### Despite a surge in circulating MBCs post-BEL initiation, their non-proliferating phenotype appears unaltered

**Figure 6.**
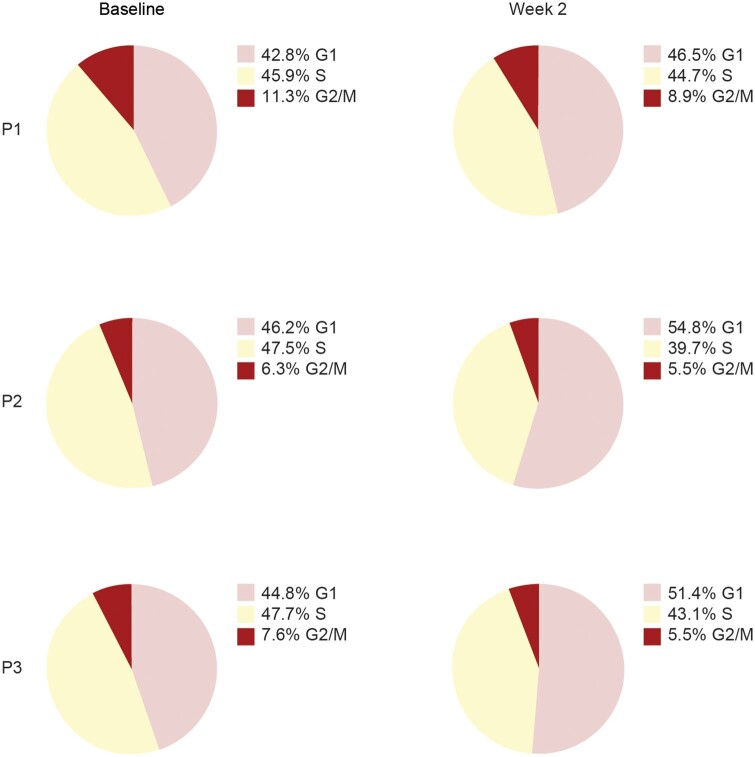
Identification of the cell cycle phase of each cell analyzed from three patients (P1, P2 and P3) with severe SLE at 2 weeks after BEL initiation, shown as percentage of MBCs per sample that are predicted to be in G1, S or G2/M phase. BEL: belimumab; MBC: memory B-cell

To investigate whether proliferation was a relevant factor for the MBC surge, we performed a cell cycle analysis to determine the phase of each cell. The analysis revealed that, at baseline, 90.5% of the measured MBCs were in the non-proliferating G1 or S phase (Fig. 6). At week 2, a decrease in proliferating G2/M phase from 9.6% to 6.5% of the total MBCs was observed. Also, functional gene expression analysis on the active proliferation marker MKI67 did not show any expression in MBCs and was not differentially expressed post-BEL initiation. Taken together, a non-proliferating phenotype of the MBCs 2 weeks post-BEL initiation was established, corresponding to the decrease in activation status found by functional enrichment analysis.

### A surge of circulating MBCs post-BEL initiation is associated with downregulation of migratory processes

Lastly, a more focused analysis on migration-associated DEGs was performed. We identified 57 downregulated biological processes associated with 99 individual DEGs related to migration, compared with no relation to any of the upregulated processes. These migratory processes can be categorized into four groups: cell adhesion, actin cytoskeleton organization, cell migration or cell chemotaxis (Fig. 5B). Of the 10 GO terms related to cell adhesion, based on 46 downregulated genes, the most significantly downregulated process was leucocyte cell–cell adhesion. Actin cytoskeleton organization was represented by 25 GO terms based on 44 genes that showed significant downregulation of actin cytoskeleton organization, filament-based process and the Arp2/3 complex-mediated actin nucleation. Cell migration was represented by 18 GO terms; the most important downregulated processes were leucocyte migration and positive regulation of cell motility. Lastly, cell chemotaxis was represented by four GO terms based on 17 genes demonstrating downregulation of cell chemotaxis and leucocyte chemotaxis (Supplementary Table S2, available at *Rheumatology* online). Collectively, these data demonstrate elaborate suppression of cell-migration processes at the RNA level of MBCs.

## Discussion

The present study firmly established that initiation of BEL led to a substantial increase in circulating MBCs, most notably in younger patients or patients with severe, serologically active SLE/LN. The absolute circulating MBC increase was present 2 weeks after treatment initiation and was sustained at 4–8 weeks, with a gradual decline to baseline values after a period of 3–12 months. The surge in circulating MBCs was not restricted to a certain subset but occurred in all MBC subsets independently of proliferation. Instead, a strong modulation of gene expression in MBCs demonstrated in a small cohort indicated a disruption of MBC trafficking with reduced expression of cell migration genes. Collectively, the surge of circulating MBCs post-BEL initiation was associated with disrupted lymphocyte trafficking, encompassing a new therapeutic mechanism attributable to BEL.

We performed a meta-analysis of flow cytometric data from four large, randomized trials [3, 6, 16, 17], and confirmed, in the largest PBO-controlled population thus far reported, the surge of circulating MBCs post-BEL initiation, which was not observed in comparator patients with SLE treated with PBO plus standard therapy. The surge of circulating MBCs post-BEL initiation was significantly higher in children compared with adults 19–45 years of age. In agreement with findings in the literature [21], which indicate that the median number of circulating MBCs tends to be higher in healthy individuals 5–17 years of age and decreases with age, it is possible that a higher number of MBCs is available to be modulated by BEL treatment. The observed surge in circulating MBCs is not unique to SLE; BEL-treated patients with primary SS (pSS) showed an increase in peripheral MBCs within 1 week, lasting until RTX infusion at 8 weeks [22]. Also, the observed effect on MBCs is not unique to BEL as other inhibitors of B-cell receptor-signalling pathways display similar effects [23–25]. For example, patients with chronic lymphocytic leukaemia treated with ibrutinib, a B-cell receptor-associated kinase inhibitor, experienced blood lymphocytosis 1 week after treatment initiation, which was reversible upon temporary drug deprivation and lasted until week 4 before a decline [23]. Patients treated with tabalumab, a humanized anti-BAFF monoclonal antibody, also experienced increases in total B cells as early as 1 week [24]. Collectively, it can be concluded that inhibition of BAFF by BEL initiation causes a surge of MBCs in the circulation.

HSFC for patients with SLE included in the randomized, controlled BLISS-BELIEVE and SynBioSe-2 trials also detected a surge of circulating MBCs. Although the use of concomitant immunosuppression could confound these analyses, the increase in MBCs was not as prominent in PBO-treated patients who were also exposed to concomitant immunosuppression. We observed a difference in amplitude of B-cell dynamics between the patients with mild/moderate SLE from the BLISS-BELIEVE trial and those with severe SLE/LN from the SynBioSe-2 trial, which might be explained by a higher steroid dosage, a greater disease severity between non-renal SLE and LN, a potential qualitative difference between LN and other SLE manifestations, or a combination of all these factors. However, as the flow cytometry data from the meta-analysis presented here also indicated a significantly greater increase in MBCs in patients with higher disease activity, it is plausible that the observed differences in MBC increases between the two trials are due to the differences in disease severity at baseline. Since BEL has good clinical efficacy, especially in patients with autoantibody-positivity and high disease activity [26], we speculate that the mobilization of MBCs to the blood could correlate with therapeutic efficacy in patients with the most severe SLE and should be further investigated as a predictive marker of response.

After confirming the surge of MBCs, which was independent of proliferation, we wanted to further determine the underpinning mechanism of the MBC response upon BEL initiation by scRNA-seq. In this study, the number of downregulated genes associated with migration processes may be explained by the lowered activation of the immune response, as B cells can regulate their location and movement according to their activation state [27]. BAFF increased B-cell chemotaxis *in vitro*, which was abolished by blockage of the BAFF-R; this B-cell chemotaxis was strongly dependent on the activation of NF-κB [28]. Ibrutinib, which blocks the downstream effects of the B-cell receptor, has also been shown to inhibit cell chemotaxis together with the inhibition of actin polymerization and activation of B-cell receptor, C–X–C motif chemokine ligand 12 (CXCL12) and CXCL13 signalling [29]. In patients with pSS, reduction of serum CXCL13 post-BEL treatment was observed upon the earliest measurement at 8 weeks [22]. The receptor for CXCL13 (C–X–C chemokine receptor type 5) is widely expressed by B cells, including MBCs. Besides its role in cell migration, CXCL13 is important in the direction of B cells into the follicles of secondary lymphoid organs and maintenance of ectopic tertiary lymphoid structures [30]. In patients with active SLE, large titres of autoantibodies are produced through high numbers of antibody-secreting cells derived from either pre-existing MBCs or germinal centre (GC) and extrafollicular (EF) reactions [31, 32], which are sites of rapid B-cell proliferation in response to different types of immunization [33]. In our study, we observed reduced gene expression levels of CD40, CCR7 and SELL (CD62L), which potentially leads to a decreased interaction with surrounding cells, e.g., dendritic and T cells, a process that is critical for the formation of GC or EF reactions [34–36]. Also, the role of BAFF in adhesion was previously established *in vitro*; high BAFF levels increase cell adhesion together with a decrease in cell adhesion on blockage of BAFF-R [37]. Additionally, previous studies have postulated the importance of BAFF in the formation of GC-like follicles, as observed in autoimmune diseases [28]. Disruption of these GC/EF reactions by BAFF deprivation, combined with an increase in circulating MBCs that are unable to migrate out of the circulation due to reduced migration capacity, is an attractive hypothetical explanation for the MBC surge. This hypothesis is further supported by histological stainings from Cynomolgus monkeys treated with BEL for 13–26 weeks, which demonstrated a decrease in CD20+ B-lymphocyte representation in lymph tissue and a decreased lymphoid follicle size and/or number [38]. With these study results, although limited by small patient numbers (*n* = 3), we can deduct that upon BEL initiation, MBCs are de-activated, non-proliferative and demonstrate characteristics of disrupted lymphocyte trafficking residing in the peripheral blood of patients with SLE.

The findings presented here have important implications for our understanding and subsequent improvement of B-cell targeted treatment strategies in patients with active SLE and LN. MBC accumulation in circulation might allow for more efficient targeting of the B-cell compartment as significant fractions of MBCs have been shown to escape from anti-CD20 treatment [39], likely due to the localization in lymphoid tissues or their survival sites [14]. Although RTX treatment results in nearly complete depletion of CD20+ B cells in the peripheral blood [40], relatively high numbers of CD20+ B cells can persist in lymphoid organs such as the bone marrow and lymph nodes [41, 42]. Furthermore, B cells have also been shown to persist in target tissues in autoimmune diseases, such as in the synovium tissue in rheumatoid arthritis [43] and in salivary glands in patients with pSS [44]. The relative resistance of tissue-resident CD20+ B cells to the effects of RTX may be related to sustained BAFF signalling [45]. The sequential therapy of BEL followed by anti-CD20 therapy for the treatment of severe SLE is promising and awaits further data from ongoing clinical trials [18, 19].

A potential limitation of the study was that the meta-analysis was performed using existing data generated from flow cytometry study designs that were not identical, as the assay and gating approaches between the different clinical studies evolved over the 15 study years. Despite this, the kinetics of circulating MBCs following BEL administration were generally consistent across studies. In support of observing robust modulation of MBC levels across the different clinical studies, MBCs are a relatively abundant B-cell subset and further increases in MBCs observed following BEL initiation greatly enhance the accurate identification and enumeration of this B-cell subset by flow cytometry. Another limitation was that the transcriptomic analysis was unable to firmly establish whether a specific tissue-derived MBC subset migrated into the circulation. The absence of evidence for this subset could be explained by the downregulation of tissue-specific markers upon entering the circulation. Alternatively, it might take longer for BEL to reach sufficient levels in the tissue to affect tissue-resident MBCs. Another limitation is that the transcriptomic data are based on a small number of patient samples and the DEG data were investigated within the same patient over time; by not introducing comparisons with healthy controls, the potential introduction of heterogeneity associated with DEG in different individuals was minimized. These analyses included classical CD20+CD27+ MBC markers, which possibly excluded atypical MBCs. Also, all analyses are based on peripheral blood, which limits the insights on complete MBC population kinetics that ideally would have been gained by analysis of MBCs in tissue. Consequently, although well-placed in the literature, we remain dependent on previously obtained transcriptomic data interpretations. For example, BEL-treated patients with systemic sclerosis showed a significant decrease in the expression of B-cell signalling in the skin, established by DNA microarray [46]. The transcriptomic changes in this trial can be attributed to a direct effect of BEL due to the significant decrease in activity of the downstream pathway of BAFF indicated by the observed reduction in TNF-mediated and NIK/NF-κB signalling pathway [47]. Furthermore, the downregulation of the interferon type I signature, which is implicated in the pathogenesis of SLE [48], and the downregulation of the Fc receptor-signalling pathway together indicate a specific change in autoimmune phenotype, as autoantibodies and immune complexes have been shown to lead to inflammation through Fc receptor crosslinking [49], thus indicating an effective initial treatment.

MBCs can contribute to autoimmune diseases through various mechanisms, including presentation of autoantigens, production of autoantibodies or cytokines, or formation of germinal centres [50]. Here, a substantial increase in circulating MBCs, particularly in patients with severe, serologically active SLE/LN, was firmly established upon BEL initiation. The increase suggests reduced MBC levels in the tissue leading to MBCs no longer interacting effectively with other immune cells, such as T cells, in lymphoid structures, possibly compromising the immune responses. The surge of circulating MBCs associated with disrupted lymphocyte trafficking of MBCs, thus, expands the existing understanding of the therapeutic mechanism of BEL’s impact on MBCs in SLE that might allow for more efficient targeting of the B-cell compartment.

## Supplementary Material

keae286_Supplementary_Data

## Data Availability

Anonymized patient-level data and study documents from individual studies can be requested for further research from www.clinicalstudydatarequest.com.

## References

[1] Anolik JH. B cell biology: implications for treatment of systemic lupus erythematosus. Lupus 2013;22:342–9. 23553777 10.1177/0961203312471576

[2] Levy RA , Gonzalez-Rivera T, Khamashta M et al 10 Years of belimumab experience: what have we learnt? Lupus 2021;30:1705–21. 34238087 10.1177/09612033211028653 PMC8564244

[3] Furie R , Petri M, Zamani O et al A phase III, randomized, placebo-controlled study of belimumab, a monoclonal antibody that inhibits B lymphocyte stimulator, in patients with systemic lupus erythematosus. Arthritis Rheum 2011;63:3918–30. 22127708 10.1002/art.30613 PMC5007058

[4] Rauch M , Tussiwand R, Bosco N, Rolink AG. Crucial role for BAFF-BAFF-R signaling in the survival and maintenance of mature B cells. PLoS One 2009;4:e5456. 19421318 10.1371/journal.pone.0005456 PMC2673681

[5] Chatham WW , Wallace DJ, Stohl W et al Effect of belimumab on vaccine antigen antibodies to influenza, pneumococcal, and tetanus vaccines in patients with systemic lupus erythematosus in the BLISS-76 trial. J Rheumatol 2012;39:1632–40. 22707609 10.3899/jrheum.111587

[6] Wallace DJ , Stohl W, Furie RA et al A phase II, randomized, double-blind, placebo-controlled, dose-ranging study of belimumab in patients with active systemic lupus erythematosus. Arthritis Rheum 2009;61:1168–78. 19714604 10.1002/art.24699 PMC2758229

[7] Stohl W , Hiepe F, Latinis KM et al Belimumab reduces autoantibodies, normalizes low complement levels, and reduces select B cell populations in patients with systemic lupus erythematosus. Arthritis Rheum 2012;64:2328–37. 22275291 10.1002/art.34400 PMC3350827

[8] Vital EM , Dass S, Buch MH et al B cell biomarkers of rituximab responses in systemic lupus erythematosus. Arthritis Rheum 2011;63:3038–47. 21618204 10.1002/art.30466

[9] Adlowitz DG , Barnard J, Biear JN et al Expansion of activated peripheral blood memory B cells in rheumatoid arthritis, impact of B cell depletion therapy, and biomarkers of response. PLoS One 2015;10:e0128269. 26047509 10.1371/journal.pone.0128269 PMC4457888

[10] Rovin BH , Furie R, Latinis K et al Efficacy and safety of rituximab in patients with active proliferative lupus nephritis: the lupus nephritis assessment with rituximab study. Arthritis Rheum 2012;64:1215–26. 22231479 10.1002/art.34359

[11] Merrill JT , Neuwelt CM, Wallace DJ et al Efficacy and safety of rituximab in moderately-to-severely active systemic lupus erythematosus: the randomized, double-blind, phase II/III systemic lupus erythematosus evaluation of rituximab trial. Arthritis Rheum 2010;62:222–33. 20039413 10.1002/art.27233 PMC4548300

[12] Kraaij T , Arends EJ, van Dam LS et al Long-term effects of combined B-cell immunomodulation with rituximab and belimumab in severe, refractory systemic lupus erythematosus: 2-year results. Nephrol Dial Transplant 2021;36:1474–83. 32591783 10.1093/ndt/gfaa117 PMC8311580

[13] Akkaya M , Kwak K, Pierce SK. B cell memory: building two walls of protection against pathogens. Nat Rev Immunol 2020;20:229–38. 31836872 10.1038/s41577-019-0244-2 PMC7223087

[14] Nguyen DC , Duan M, Ali M et al Plasma cell survival: the intrinsic drivers, migratory signals, and extrinsic regulators. Immunol Rev 2021;303:138–53. 34337772 10.1111/imr.13013 PMC8387437

[15] Teng YKO , Wheater G, Hogan VE et al Induction of long-term B-cell depletion in refractory rheumatoid arthritis patients preferentially affects autoreactive more than protective humoral immunity. Arthritis Res Ther 2012;14:R57. 22409963 10.1186/ar3770 PMC3446423

[16] Ginzler E , Guedes Barbosa LS, D'Cruz D et al Phase III/IV, randomized, fifty-two-week study of the efficacy and safety of belimumab in patients of Black African ancestry with systemic lupus erythematosus. Arthritis Rheumatol 2022;74:112–23. 34164944 10.1002/art.41900 PMC9300099

[17] Brunner HI , Abud-Mendoza C, Viola DO et al Safety and efficacy of intravenous belimumab in children with systemic lupus erythematosus: results from a randomised, placebo-controlled trial. Ann Rheum Dis 2020;79:1340–8. 32699034 10.1136/annrheumdis-2020-217101 PMC7509523

[18] Teng YKO , Bruce IN, Diamond B et al Phase III, multicentre, randomised, double-blind, placebo-controlled, 104-week study of subcutaneous belimumab administered in combination with rituximab in adults with systemic lupus erythematosus (SLE): BLISS-BELIEVE study protocol. BMJ Open 2019;9:e025687. 10.1136/bmjopen-2018-025687 PMC6475247 30898822

[19] van Schaik M , Arends EJ, Soonawala D et al Efficacy of belimumab combined with rituximab in severe systemic lupus erythematosus: study protocol for the phase 3, multicenter, randomized, open-label Synbiose 2 trial. Trials 2022;23:939. 36371234 10.1186/s13063-022-06874-w PMC9652788

[20] ClinicalTrials.gov. Synergetic B-cell immodulation in SLE – 1st study. (SynBioSe-1). 2019. https://clinicaltrials.gov/study/NCT02284984 (December 2023, date last accessed).

[21] Blanco E , Pérez-Andrés M, Arriba-Méndez S et al Age-associated distribution of normal B-cell and plasma cell subsets in peripheral blood. J Allergy Clin Immunol 2018;141:2208–19.e16. 29505809 10.1016/j.jaci.2018.02.017

[22] Mariette X , Barone F, Baldini C et al A randomized, phase II study of sequential belimumab and rituximab in primary Sjogren's syndrome. JCI Insight 2022;7:e163030. 36477362 10.1172/jci.insight.163030 PMC9746921

[23] Byrd JC , Furman RR, Coutre SE et al Targeting BTK with ibrutinib in relapsed chronic lymphocytic leukemia. N Engl J Med 2013;369:32–42. 23782158 10.1056/NEJMoa1215637 PMC3772525

[24] Isenberg DA , Petri M, Kalunian K et al Efficacy and safety of subcutaneous tabalumab in patients with systemic lupus erythematosus: results from ILLUMINATE-1, a 52-week, phase III, multicentre, randomised, double-blind, placebo-controlled study. Ann Rheum Dis 2016;75:323–31. 26338095 10.1136/annrheumdis-2015-207653

[25] Stohl W , Merrill JT, Looney RJ et al Treatment of systemic lupus erythematosus patients with the BAFF antagonist "peptibody" blisibimod (AMG 623/A-623): results from randomized, double-blind phase 1a and phase 1b trials. Arthritis Res Ther 2015;17:215. 26290435 10.1186/s13075-015-0741-z PMC4545922

[26] Oon S , Huq M, Golder V et al Lupus low disease activity state (LLDAS) discriminates responders in the BLISS-52 and BLISS-76 phase III trials of belimumab in systemic lupus erythematosus. Ann Rheum Dis 2019;78:629–33. 30679152 10.1136/annrheumdis-2018-214427

[27] Carrasco YR , Batista FD. B cell recognition of membrane-bound antigen: an exquisite way of sensing ligands. Curr Opin Immunol 2006;18:286–91. 16616474 10.1016/j.coi.2006.03.013

[28] Badr G , Borhis G, Lefevre EA et al BAFF enhances chemotaxis of primary human B cells: a particular synergy between BAFF and CXCL13 on memory B cells. Blood 2008;111:2744–54. 18172003 10.1182/blood-2007-03-081232

[29] Ponader S , Chen S-S, Buggy JJ et al The bruton tyrosine kinase inhibitor PCI-32765 thwarts chronic lymphocytic leukemia cell survival and tissue homing in vitro and in vivo. Blood 2012;119:1182–9. 22180443 10.1182/blood-2011-10-386417 PMC4916557

[30] Henneken M , Dörner T, Burmester G-R, Berek C. Differential expression of chemokine receptors on peripheral blood B cells from patients with rheumatoid arthritis and systemic lupus erythematosus. Arthritis Res Ther 2005;7:R1001–13. 16207316 10.1186/ar1776 PMC1257429

[31] Tipton CM , Fucile CF, Darce J et al Diversity, cellular origin and autoreactivity of antibody-secreting cell population expansions in acute systemic lupus erythematosus. Nat Immunol 2015;16:755–65. 26006014 10.1038/ni.3175 PMC4512288

[32] Vinuesa CG , Sanz I, Cook MC. Dysregulation of germinal centres in autoimmune disease. Nat Rev Immunol 2009;9:845–57. 19935804 10.1038/nri2637

[33] Suurmond J , Atisha-Fregoso Y, Barlev AN et al Patterns of ANA+ B cells for SLE patient stratification. JCI Insight 2019;4:e127885. 31045579 10.1172/jci.insight.127885 PMC6538361

[34] Ise W , Fujii K, Shiroguchi K et al T follicular helper cell-germinal center B cell interaction strength regulates entry into plasma cell or recycling germinal center cell fate. Immunity 2018;48:702–15.e4. 29669250 10.1016/j.immuni.2018.03.027

[35] Gatto D , Wood K, Brink R. EBI2 operates independently of but in cooperation with CXCR5 and CCR7 to direct B cell migration and organization in follicles and the germinal center. J Immunol 2011;187:4621–8. 21948984 10.4049/jimmunol.1101542

[36] Lagresle C , Bella C, Daniel P, Krammer P, Defrance T. Regulation of germinal center B cell differentiation. Role of the human APO-1/Fas (CD95) molecule. J Immunol 1995;154:5746–56. 7538529

[37] Tai Y-T , Li X-F, Breitkreutz I et al Role of B-cell-activating factor in adhesion and growth of human multiple myeloma cells in the bone marrow microenvironment. Cancer Res 2006;66:6675–82. 16818641 10.1158/0008-5472.CAN-06-0190

[38] Halpern WG , Lappin P, Zanardi T et al Chronic administration of belimumab, a BLyS antagonist, decreases tissue and peripheral blood B-lymphocyte populations in cynomolgus monkeys: pharmacokinetic, pharmacodynamic, and toxicologic effects. Toxicol Sci 2006;91:586–99. 16517838 10.1093/toxsci/kfj148

[39] van Dam LS , Oskam JM, Kamerling SWA et al Highly sensitive flow cytometric detection of residual B-cells after rituximab in anti-neutrophil cytoplasmic antibodies-associated vasculitis patients. Front Immunol 2020;11:566732. 33384685 10.3389/fimmu.2020.566732 PMC7770159

[40] Leandro MJ , Cambridge G, Ehrenstein MR, Edwards JC. Reconstitution of peripheral blood B cells after depletion with rituximab in patients with rheumatoid arthritis. Arthritis Rheum 2006;54:613–20. 16447239 10.1002/art.21617

[41] Rehnberg M , Amu S, Tarkowski A, Bokarewa MI, Brisslert M. Short- and long-term effects of anti-CD20 treatment on B cell ontogeny in bone marrow of patients with rheumatoid arthritis. Arthritis Res Ther 2009;11:R123. 19686595 10.1186/ar2789 PMC2745807

[42] Ramwadhdoebe TH , van Baarsen LGM, Boumans MJH et al Effect of rituximab treatment on T and B cell subsets in lymph node biopsies of patients with rheumatoid arthritis. Rheumatology 2019;58:1075–85. 30649469 10.1093/rheumatology/key428 PMC6532448

[43] Teng YK , Levarht EW, Toes RE, Huizinga TW, van Laar JM. Residual inflammation after rituximab treatment is associated with sustained synovial plasma cell infiltration and enhanced B cell repopulation. Ann Rheum Dis 2009;68:1011–6. 18647852 10.1136/ard.2008.092791

[44] Pijpe J , Meijer JM, Bootsma H et al Clinical and histologic evidence of salivary gland restoration supports the efficacy of rituximab treatment in Sjögren’s syndrome. Arthritis Rheum 2009;60:3251–6. 19877054 10.1002/art.24903

[45] Gong Q , Ou Q, Ye S et al Importance of cellular microenvironment and circulatory dynamics in B cell immunotherapy. J Immunol 2005;174:817–26. 15634903 10.4049/jimmunol.174.2.817

[46] Gordon JK , Martyanov V, Franks JM et al Belimumab for the treatment of early diffuse systemic sclerosis: results of a randomized, double-blind, placebo-controlled, pilot trial. Arthritis Rheumatol 2018;70:308–16. 29073351 10.1002/art.40358 PMC6590997

[47] Pflug KM , Sitcheran R. Targeting NF-kappaB-inducing kinase (NIK) in immunity, inflammation, and cancer. Int J Mol Sci 2020;21:8470. 33187137 10.3390/ijms21228470 PMC7696043

[48] Brohawn PZ , Streicher K, Higgs BW et al Type I interferon gene signature test-low and -high patients with systemic lupus erythematosus have distinct gene expression signatures. Lupus 2019;28:1524–33. 31660791 10.1177/0961203319885447

[49] Ben Mkaddem S , Benhamou M, Monteiro RC. Understanding Fc receptor involvement in inflammatory diseases: from mechanisms to new therapeutic tools. Front Immunol 2019;10:811. 31057544 10.3389/fimmu.2019.00811 PMC6481281

[50] Hampe CS. B cell in autoimmune diseases. Scientifica 2012;2012:215308. 23807906 10.6064/2012/215308 PMC3692299

