# Supplementary material for: Disruption of memory B-cell trafficking by belimumab in patients with systemic lupus erythematosus
Source: Rheumatology (Oxford). 2024 May 22;63(9):2387–98. doi: 10.1093/rheumatology/keae286 (PMC11371378; doi:10.1093/rheumatology/keae286)
Supplement: keae286_Supplementary_Data [file keae286_supplementary_data.pdf]

**Disruption of Memory B-cell Trafficking by Belimumab in Patients with Systemic Lupus Erythematosus (99/140 characters, including spaces)**

**Authors:** Eline J. Arends, MD[1], Mihaela Zlei, PhD[2, 3]*, Christopher M. Tipton, PhD[4, 5], Jasna Cotic, PhD[6], Zgjim Osmani, MSc[1], Fenna J. de Bie, MSc[2], Sylvia W.A. Kamerling, BASc[1]*, Andre van Maurik, PhD[7], Richard Dimelow, PhD[8], Yun Irene Gregan, MD[9]*, Norma Lynn Fox, PhD[10]*, Ton J. Rabelink, MD, PhD[1], David A. Roth, MD[11], Ignacio Sanz, MD[4], Jacques J.M. van Dongen, MD, PhD[12], Cees van Kooten, PhD[1], Y.K. Onno Teng, MD, PhD[1†]

*At the time of the study.

**Affiliations:** [1]Expert Center for Lupus-, Vasculitis-, and Complement-mediated Systemic diseases (LuVaCs), Department of Internal Medicine – section Nephrology, Leiden University Medical Centre; Leiden, The Netherlands; [2]Department of Immunology, Leiden University Medical Centre; Leiden, The Netherlands; [3]Medical Laboratory, Department of Flow Cytometry, Regional Institute of Oncology; Iasi, Romania; [4]Lowance Centre for Human Immunology, Emory University School of Medicine; Atlanta, GA, USA; [5]Department of Medicine, Division of Rheumatology, Emory University; Atlanta, GA, USA; [6]Clinical Statistics, GSK; Brentford, Middlesex, UK; [7]Clinical Pharmacology and Experimental Medicine, GSK; Stevenage, Hertfordshire, UK; [8]Clinical Pharmacology Modelling and Simulation, GSK; Stevenage, Hertfordshire, UK; [9]Clinical Science Immunology, GSK; Collegeville, PA, USA; [10]Clinical Development, GSK; Collegeville, PA, USA; [11]Research and Development, GSK; Collegeville, PA, USA; [12]Centro de Investigación del Cáncer-Instituto de Biología Molecular y Celular del Cáncer (CIC-IBMCC, USAL-CSIC-FICUS) and Department of Medicine, University of Salamanca; Salamanca, Spain.

†**Corresponding author:** Y.K. Onno Teng

**Address:** Department of Nephrology, Leiden University Medical Centre (LUMC), P.O. Box 9600, 2300 RC Leiden, The Netherlands

**Email:** y.k.o.teng@lumc.nl

ORCID ID: 0000-0001-9920-2195

**Target journal:** *Rheumatology*

**Article Type:** Original article

**Word count:** 3865/3500

**Figures/Tables:** 6/6 figures (5 colour figures)

**References:** Currently 50/50

**Supplementary materials:** Supplementary methods, 4 figures and 2 tables

## SUPPLEMENTARY MATERIAL

### 1. Supplementary methods

#### 1.1. Retrospective meta-analysis of flow cytometry data: covariate analysis

Memory B cell (MBC) percentage change from baseline at Week 8 was analysed for univariate associations with age, race, B-cell–activating factor (BAFF) levels, systemic lupus erythematosus (SLE) Disease Activity Index (SLEDAI) indices score (Safety of Estrogens in Lupus National Assessment [SELENA]-SLEDAI was used in LBSL02, BLISS-76, and PLUTO; SLEDAI-2K was used in EMBRACE), complement levels, anti-dsDNA antibody status, and usage of SLE standard therapy (steroids, immunosuppressants, antimalarials), as reported at baseline.

#### 1.2. EuroFlow-based B-cell subset high-sensitivity flow cytometry (HSFC)

Peripheral blood was collected in ethylenediaminetetraacetic acid (EDTA)-coated tubes at baseline and 4 weeks after treatment initiation with belimumab (BEL). Samples were processed within 4 hours (SynBioSe-2 trial (1)) and 33 hours (BLISS-BELIEVE (2)) after collection. All samples were analysed by HSFC with the BD LSRFortessa X-20 4L (BD Biosciences) flow cytometer (Flow cytometry Core Facility, Leiden University Medical Center [LUMC]) after bulk-lyse standard operating procedure (www.EuroFlow.org), as described previously (3, 4). Per patient sample, the surface membrane (sm) of $20\times10^6$ nucleated cells was stained with the EuroFlow Immunoglobulin (Ig)H-isotype B-cell antibody combination and 10–$20\times10^6$ leukocytes were measured. Instrument set-up and calibration were performed according to EuroFlow standard operating procedures (5). For data analysis, Infinicyt software version 2.1.0 (Cytognos S.L., Salamanca, Spain) was used. The gating strategy, performed as previously shown (3, 4, 6), was used for the identification

of different B-cell subsets and MBC populations (CD19+, CD27+, $CD38^{lo}$, $CD24^{het}$), which were further subclassified according to the Ig isotypes/subsets (smIgM+, smIgD+, smIgG1+, smIgG2+, smIgG3+, smIgG4+, smIgA1+, smIgA2+). B-cell subsets are classified according to the following definitions: immature (CD27-$CD38^{hi}CD24^{hi}$CD5+smIgM++IgD+), naïve (CD27-$CD38^{lo}CD24^{het}$CD5+smIgM+IgD++), mature naïve (CD27-$CD38^{lo}CD24^{het}$CD5-smIgM+IgD++), unswitched memory (CD27+$CD38^{lo}CD24^{het}$smIgM++IgD+), switched memory (CD27+$CD38^{lo}CD24^{het}$smIgM-IgD-), and plasma ($CD27^{hi}CD38^{hi}$CD21-CD24-).

**1.3. Isolation of classical MBCs by fluorescence-activated cell sorting (FACS)**

Cryopreserved peripheral blood mononuclear cell samples were thawed simultaneously for cell sorting of classical MBCs: CD45+ (2D1, PerCP, BD Biosciences, San Jose, CA), CD3- (SK7, FITC, BD Biosciences, San Jose, CA), CD20+ (2H7, PE, eBiosciences, San Diego, CA), CD27+ (LG.7F9, PeCy7, Thermo Fischer Scientific, Waltham, MA). Before cell staining, cells were incubated with FcX blocking agent (TrueStain FcXTM, BioLegend). During cell staining, each sample was labelled with a unique hashing antibody (0.5 $\mu g/2\times10^6$ cells) to enable multiplexing of samples. Oligo-tagged monoclonal hashtag antibodies directed against the most broadly expressed surface proteins in human tissues (i.e., ß-2 microglobulin and CD298) were used, each containing its own traceable unique oligonucleotide sequence (TotalSeq, clone 2M2 LHH.94 mouse IgG1κ). Sorting of classical MBCs was performed with FACS Aria-III cell sorters (BD Biosciences) in the Flow cytometry Core Facility of LUMC. After sorting, MBCs were stored in Roswell Park Memorial Institute (RPMI) medium 1640 (Gibco, Paisley, UK) with 40% fetal bovine serum (Bodinco, Alkmaar, The Netherlands). Cells were transported on ice to the department of Human Genetics (Leiden Genome Technology Center [LGTC]) for single-cell sequencing library preparation.

### 1.4. Single-cell ribonucleic acid sequencing (scRNA-seq) of classical MBCs

Sorted MBCs were grouped into three pools, each containing paired samples of baseline and Week 2. If possible, live, and dead cell counting was performed before equimolar pooling of samples. Library preparation was performed for 10x Genomics single-cell RNA according to CITE-seq and Hashing protocol 190213 (cite-seq.com). After library preparation, samples were delivered to GenomeScan (Leiden BioScience Park, The Netherlands) and were sequenced on a partial lane of the Illumina NovaSeq6000 (150 base pair paired-end sequencing). Library preparation and sequencing was performed in one run to reduce technical variation. Raw sequence data met the quality requirements and the standard 10x Genomics Cell Ranger Pipeline (v3.1) was run at the department of Human Genetics (Transcriptome: GRCh38-3.0.0).

### 1.5. scRNA-seq data analysis

Gene expression data of each cell were demultiplexed to their original sample-of-origin based on hashtag enrichment. In addition, cross-sample doublets (i.e., cells expressing more than one hashtag) were identified and discarded. Low-quality cells (i.e., apoptotic cells, empty droplets) were excluded by removing cells expressing more than 5% mitochondrial genes and fewer than 250 unique genes (7). Cells expressing more than 2200 unique genes were considered to be intra-sample doublets and were therefore excluded from the analysis. Furthermore, a small number of cells (<1%) expressing monocytic (CD14, LYZ), dendritic cell (CST3), T cell (CD3E, CD3D, CD8A, CD8B), and cytotoxic T cell/natural killer cell markers (GNLY, GZMB, PRF1) were detected and excluded from further downstream analysis. A small number of naïve B cells (IgM/D+CD27-) and plasma cells (IRF4+PRDM1+XBP1+) were detected but were not excluded from analysis.

VDJ sequencing data was analysed using a software pipeline developed in-house in the Sanz lab and written in Perl (v5.18) and MATLAB (R2018b). Full methodology for the data processing, visualization, and analysis using this pipeline has been described previously (8). Sequences were reconstructed using 10x Genomics Cell Ranger software (v4.0), and resultant VDJ sequences were uploaded to IMGT/HighV-QUEST for mutation calculations and alignment analyses (9). Isotype assignment was made using Cell Ranger (v4.0) and validated using in-house software to match constant region alignments to known sequences. The frequency and distribution of somatic hypermutation were ascertained based on non-gap mismatches of expressed sequences with the closest germline VH sequence. Mutation frequencies were determined by calculating the percentage of number of V gene mutations relative to number of non-gap V gene bases. Mutation density plot and isotype composition were plotted using R (v3.6.3) (10). All clonal assignments were based on matching V and J regions, matching complementarity-determining region 3 (CDR3) length, and 85% total nucleotide similarity within the CDR3.

Single-cell gene expression data for each sample were normalized using the SCTransform normalization (11). Single-cell datasets from all samples were integrated by reference-based reciprocal PCA integration (12, 13), enabling comparison of single-cell sequence data in which baseline samples were selected as a reference for data integration. Derived log normalized counts were used for both differential gene expression and cell cycle analysis. Cell cycle analysis was performed to determine the cell cycle phase of each cell by assigning a cell cycle score to each cell based on its expression of several different G2/M and S phase markers. The cell cycle phase of each cell was predicted based on the cell cycle score; cells that did not express any G2/M or S phase genes were considered to be in G1 phase. Differentially expressed genes (DEGs) were identified by a non-parametric Wilcoxon rank-sum test using a filter requiring at least an average $\log_2$ fold change of 0.25, adjusted p-value

<0.05 and ≥10% of the cells expressing the gene of interest. Functional enrichment analysis (FEA) was performed using the gene ontology database looking at biological processes (14). FEA results were also used for the migration analysis selecting all biological processes associated with cell adhesion, actin cytoskeleton organization, cell migration, or cell chemotaxis, for which the corresponding DEG and average $\log_2$ fold changes were assessed.

**1.6. Statistics**

Continuous covariates were assessed with Spearman's Rank correlation coefficient, with adjustments for the following baseline characteristics: age, SLEDAI indices score, BAFF levels, or anti-dsDNA antibody levels. Logarithmic transformations were performed to improve visualization of data with skewed distributions. Categorical covariates were analysed with Wilcoxon rank-sum test (significance level: $\alpha=0.05$). Analyses were conducted in SAS version 9.4. Other statistical analyses were performed in GraphPad Prism (v9.0.1) and R (v3.6.3) (10) using the Seurat package (v3.1.4.9019+v4.0). Differences in absolute cell counts between time points were tested with the Wilcoxon matched-pairs signed-rank test, and differences in fold change between groups were tested using the Mann-Whitney method. For DEG analysis, non-parametric Wilcoxon rank-sum test was used, and significance was defined as $p<0.05$ after applying the Bonferroni correction for multiple testing.

## 2. SUPPLEMENTARY FIGURES AND TABLES

**Supplementary Figure S1. Correlation plots with Spearman's Rank correlation coefficients for the associations of percentage change in MBCs (cells/µL) from baseline at Week 8 with age, anti-dsDNA levels, BAFF levels and SLEDAI* score in patients receiving BEL 10 mg/kg IV or PBO in four pooled SLE clinical trials.**

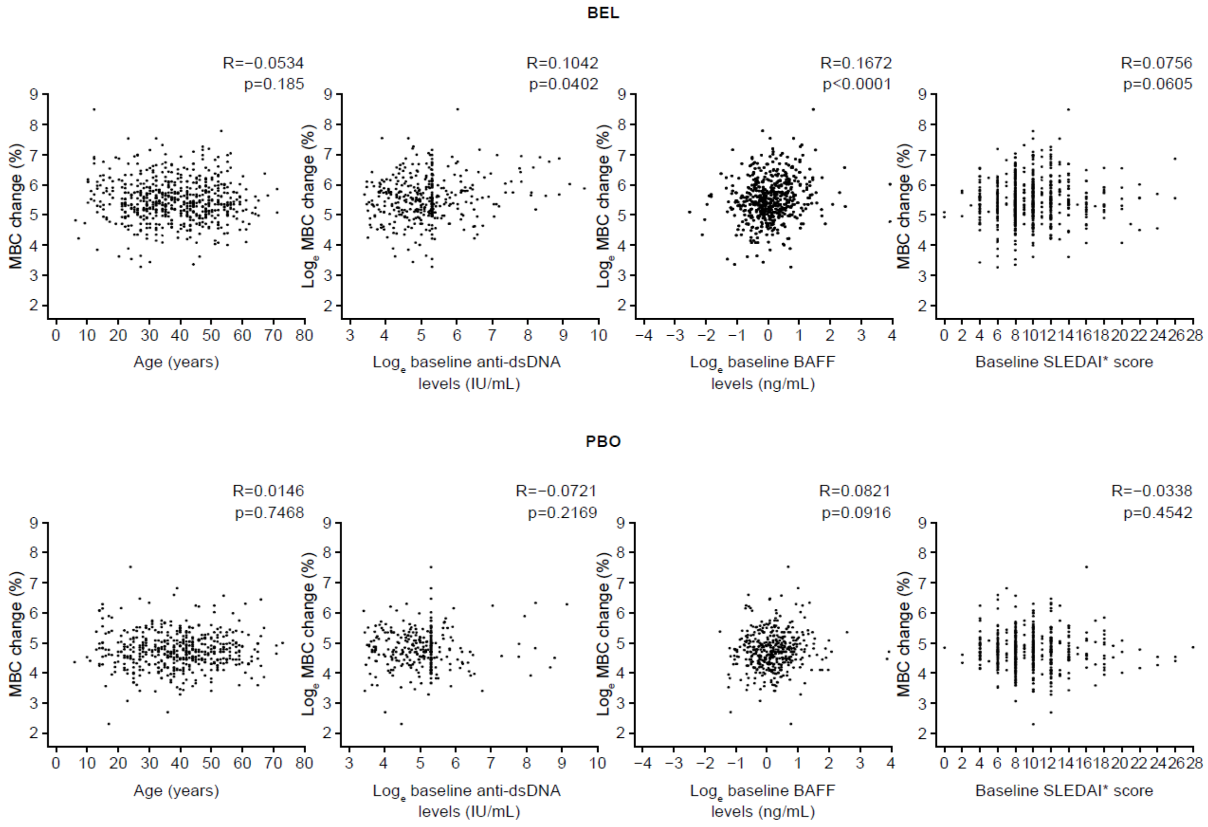


Logarithmic transformations were applied to improve visualization of data with skewed distributions.

*SELENA-SLEDAI was used in studies LBSL02, BLISS-76, and PLUTO; SLEDAI-2K was used in EMBRACE.

BAFF, B-cell–activating factor; BEL, belimumab; anti-dsDNA, anti-double-stranded deoxyribonucleic acid antibody; IV, intravenous; MBC, memory B cell; PBO, placebo; R, Spearman's Rank correlation coefficient; SELENA-SLEDAI, Safety of Estrogens in Lupus

National Assessment - SLE Disease Activity Index; SLE, systemic lupus erythematosus; SLEDAI–2K, SLE Disease Activity Index 2000.

**Supplementary Figure S2. B-cell changes in patients with severely active SLE/LN after initiation of BEL measured by high-sensitivity FACS by EuroFlow protocol. Depicted are changes in B-cell subsets (A, C) and MBC Ig subsets (B, D) at baseline compared with Week 2 in absolute cell counts (A, B) and in median (IQR) percentage changes (C, D).**

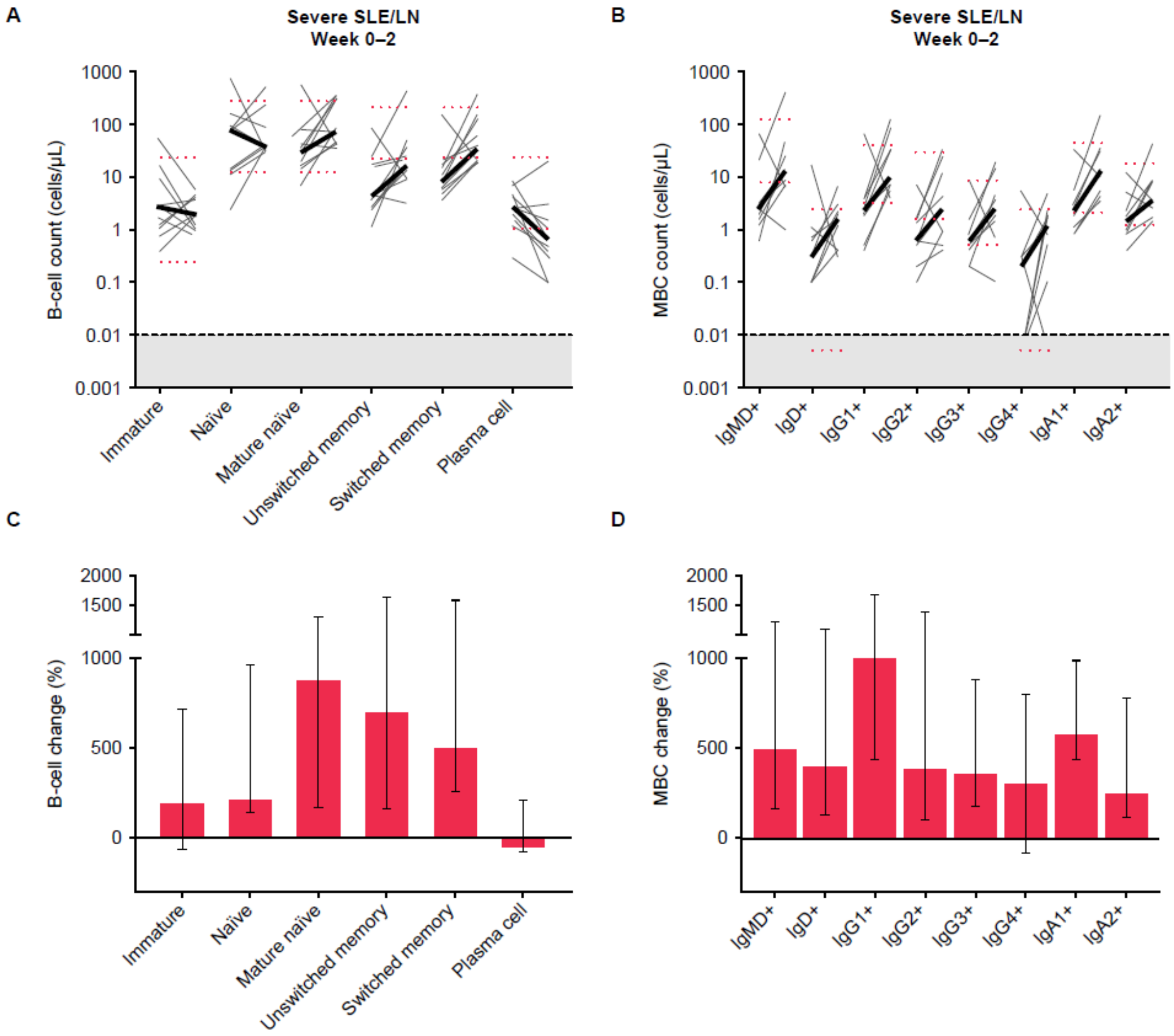


Note: The grey area indicates 1–20 analysed events that were excluded from the analysis. Black dotted line at 0.01 cells/µL indicates the detection limit. Red dotted lines indicate the proposed normal values for adults 18–59 years of age (3).

BEL, belimumab; FACS, fluorescence-activated cell sorting; Ig, immunoglobulin; IQR, interquartile range; LN, lupus nephritis; MBC, memory B cell; SLE, systemic lupus erythematosus.

**Supplementary Figure S3. Changes in sequence density and mutation frequencies of B-cell Ig isotypes with the use of scRNA-seq on sorted MBCs from three patients (P1, P2, and P3) with severe SLE/LN comparing baseline with 2 weeks after initiation of BEL (A). Distribution of the different Ig subsets at baseline and 2 weeks after initiation of BEL (B).**

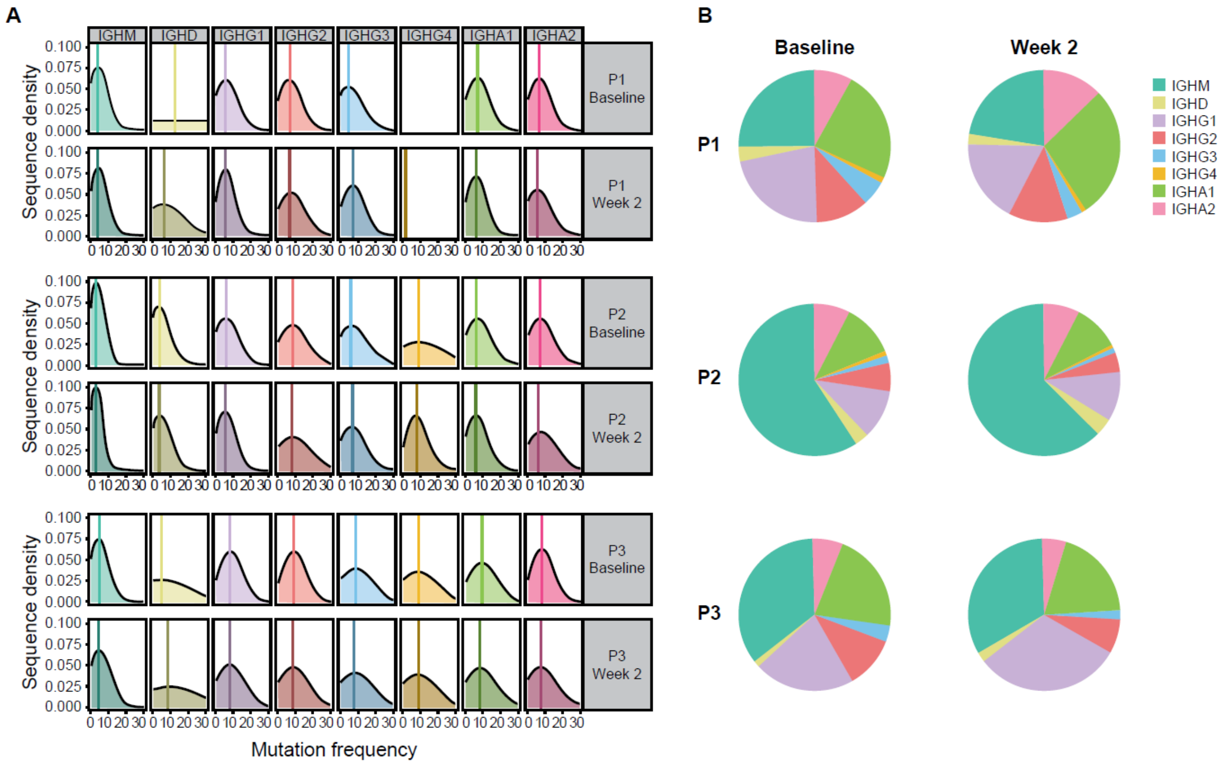


BEL, belimumab; Ig, immunoglobulin; IGHA, immunoglobulin heavy constant alpha; IGHD, immunoglobulin heavy constant delta; IGHG, immunoglobulin heavy constant gamma; IGHM, immunoglobulin heavy constant mu; MBC, memory B cells; scRNA-seq, single-cell ribonucleic acid sequencing; SLE, systemic lupus erythematosus.

**Supplementary Figure S4. Identification of co-DEGs at 2 weeks after BEL initiation. Depicted are genes with a minimal percentage expression of 10% and a change above 0.25 in all three analysed patients (P1, P2, and P3) with severe SLE.**

Co-DEGs (n=102)

| | P1 | P2 | P3 |
|---|---|---|---|
| NFKBIA | −1.4 | −0.5 | −2.6 |
| IFI44L | −2.0 | −0.5 | −1.3 |
| LTB | −1.2 | −1.4 | −0.9 |
| XAF1 | −1.9 | −0.4 | −1.0 |
| MX1 | −1.4 | −0.6 | −1.0 |
| IFITM1 | −1.4 | −0.4 | −1.2 |
| IGKV3-20 | −1.3 | −1.0 | −0.7 |
| ISG15 | −1.2 | −0.4 | −1.1 |
| BIRC3 | −1.1 | −0.5 | −1.0 |
| IRF7 | −1.3 | −0.4 | −0.8 |
| OAS1 | −1.2 | −0.6 | −0.5 |
| CTSH | −0.8 | −0.7 | −0.7 |
| IER2 | −1.2 | −0.4 | −1.4 |
| STAT1 | −0.6 | −0.6 | −0.9 |
| IGKC | −1.0 | −0.8 | −0.3 |
| SELL | −0.8 | −0.9 | −0.3 |
| SUB1 | −0.9 | −0.6 | −0.6 |
| EPSTI1 | −1.0 | −0.4 | −0.6 |
| BST2 | −1.2 | −0.3 | −0.4 |
| CD69 | −0.7 | 0.5 | −1.8 |
| PLEK | −0.7 | −0.6 | −0.6 |
| CD83 | −0.3 | 0.7 | −2.3 |
| RAC2 | −0.8 | −0.6 | −0.4 |
| DYNLL1 | −0.9 | −0.4 | −0.4 |
| NFKB2 | −0.7 | −0.3 | −0.7 |
| UBE2L6 | −0.8 | −0.5 | −0.5 |
| JPT1 | −0.8 | −0.5 | −0.4 |
| HLA-A | −0.7 | −0.5 | −0.4 |
| SAMD9L | −0.7 | −0.4 | −0.5 |
| HLA-DQA1 | −0.6 | −0.6 | −0.5 |
| DNAJA1 | −0.8 | 0.3 | −1.1 |
| HSPA8 | −0.8 | −0.5 | −0.3 |
| LINC00926 | −0.5 | −0.7 | −0.5 |
| PSME2 | −0.5 | −0.5 | −0.6 |

| | P1 | P2 | P3 |
|---|---|---|---|
| TAP1 | −0.7 | −0.4 | −0.5 |
| S100A11 | −0.7 | −0.5 | −0.3 |
| CCR7 | −0.4 | −0.7 | −0.5 |
| CD40 | −0.6 | −0.6 | −0.3 |
| PRDX1 | −0.6 | −0.6 | −0.3 |
| TYMP | −0.4 | −0.4 | −0.6 |
| PSMB9 | −0.5 | −0.7 | −0.3 |
| SQSTM1 | −0.5 | −0.4 | −0.4 |
| RAN | −0.7 | −0.3 | −0.3 |
| BRK1 | −0.5 | −0.6 | −0.3 |
| TCF4 | −0.6 | −0.4 | −0.4 |
| DRAP1 | −0.4 | −0.3 | −0.6 |
| JUN | −0.6 | 0.3 | −0.9 |
| TAGAP | −0.4 | −0.3 | −0.6 |
| ERH | −0.5 | −0.5 | −0.3 |
| LYSMD2 | −0.6 | −0.4 | −0.3 |
| PPP1R18 | −0.5 | −0.4 | −0.4 |
| LIMD2 | −0.4 | −0.6 | −0.3 |
| IFI35 | −0.5 | −0.3 | −0.5 |
| BASP1 | −0.6 | −0.3 | −0.3 |
| ARPC5 | −0.4 | −0.5 | −0.3 |
| ACTR3 | −0.4 | −0.5 | −0.3 |
| SYK | −0.5 | −0.4 | −0.4 |
| LAP3 | −0.5 | −0.3 | −0.4 |
| MARCKS | −0.5 | −0.3 | −0.4 |
| ANXA4 | −0.5 | −0.4 | −0.3 |
| CHMPS | −0.5 | −0.3 | −0.3 |
| B2M | −0.6 | −0.3 | −0.3 |
| TNFRSF13B | −0.4 | −0.3 | −0.3 |
| TPI1 | −0.4 | −0.4 | −0.3 |
| GRN | −0.3 | −0.5 | −0.3 |
| TRADD | −0.5 | −0.3 | −0.3 |
| IL27RA | −0.4 | −0.3 | −0.3 |
| PPIB | −0.3 | −0.3 | −0.3 |

| | P1 | P2 | P3 |
|---|---|---|---|
| VAMP8 | −0.4 | −0.4 | −0.3 |
| GABARAPL2 | −0.4 | −0.3 | −0.3 |
| SEC61B | −0.4 | −0.3 | −0.3 |
| H3F3A | −0.3 | −0.4 | −0.3 |
| RHOH | −0.6 | 0.4 | −0.7 |
| ETFA | −0.4 | −0.3 | −0.3 |
| COX5A | −0.3 | −0.3 | −0.3 |
| PRELID1 | −0.3 | −0.3 | −0.3 |
| ABRACL | −0.3 | −0.3 | −0.3 |
| ZFP36 | −0.3 | 0.8 | −1.3 |
| FOSB | −0.3 | 0.7 | −1.2 |
| GPSM3 | −0.3 | −0.3 | −0.3 |
| DUSP2 | −0.4 | 0.8 | −1.0 |
| SRGN | −0.3 | 0.5 | −0.6 |
| CYTIP | −0.3 | 0.5 | −0.3 |
| UCP2 | 0.5 | −0.6 | 0.3 |
| PIK3IP1 | 0.3 | 0.4 | 0.4 |
| CCNH | 0.3 | 0.6 | 0.4 |
| KLF2 | 0.4 | 0.6 | 0.3 |
| RPS19 | 0.6 | 0.3 | 0.3 |
| RPS16 | 0.6 | 0.3 | 0.4 |
| PELI1 | 0.6 | 1.3 | −0.6 |
| RPS12 | 0.7 | 0.3 | 0.4 |
| SF1 | 0.3 | 0.3 | 0.8 |
| SOCS1 | 0.5 | 0.3 | 0.5 |
| RPS27A | 0.6 | 0.3 | 0.5 |
| CXCR4 | 0.8 | 1.0 | −0.4 |
| TPT1 | 0.6 | 0.3 | 0.6 |
| RPL21 | 0.8 | 0.3 | 0.5 |
| EEF1B2 | 0.7 | 0.3 | 0.7 |
| TP53INP1 | 0.7 | 0.4 | 0.7 |
| BTG1 | 0.8 | 1.0 | 0.4 |
| ZFP36L2 | 1.3 | 1.4 | 0.6 |
| TXNIP | 1.2 | 1.1 | 1.3 |

Note: Downregulation is shown in blue, upregulation in red.

BEL, belimumab; DEG, differentially expressed gene; SLE, systemic lupus erythematosus.

**Supplementary Table S1. Univariate associations of percentage change in MBCs from baseline at Week 8 after treatment initiation with categorical covariates at baseline. The analyses were conducted on pooled clinical trial data separately for each of the two treatment arms.**

| | | Placebo | | | Belimumab | | |
|---|---|---|---|---|---|---|---|
| | | n | Median % change (IQR) | p-value | n | Median % change (IQR) | p-value |
| SLEDAI* score | <10 | 241 | 0.0 (–25.0, 44.1) | | 303 | 107.1 (47.2, 216.7) | |
| | ≥10 | 251 | 0.0 (–33.3, 50.0) | 0.6151 | 315 | 133.3 (54.3, 271.4) | 0.0419 |
| Anti-dsDNA status | <30 IU/mL | 198 | –3.9 (–31.0, 31.6) | | 230 | 100.0 (42.9, 193.1) | |
| | ≥30 IU/mL | 295 | 6.7 (–28.6, 66.7) | 0.0539 | 388 | 139.4 (53.6, 280.0) | 0.0031 |
| Complement levels | Normal C3/C4 | 259 | 0.0 (–32.6, 40.4) | | 327 | 107.7 (44.4, 219.0) | |
| | Low C3 or C4 | 234 | 0.0 (–25.5, 50.0) | 0.1958 | 291 | 142.9 (55.6, 277.4) | 0.0417 |
| High serological disease activity† | No | 307 | 0.0 (–31.8, 37.5) | | 376 | 108.9 (45.1, 220.0) | |
| | Yes | 186 | 7.0 (–25.8, 60.0) | 0.0679 | 242 | 145.8 (56.0, 280.0) | 0.0314 |
| Steroid use at baseline | No | 102 | 0.0 (–20.0, 28.6) | | 144 | 104.0 (53.8, 191.5) | |
| | Yes | 391 | 0.0 (–33.3, 50.0) | 0.6558 | 474 | 126.7 (50.0, 277.4) | 0.0561 |
| Immunosuppressant use at baseline | No | 165 | 0.0 (–33.3, 57.9) | | 236 | 103.6 (46.0, 228.7) | |
| | Yes | 328 | 0.0 (–25.9, 44.3) | 0.7725 | 382 | 127.4 (55.6, 261.5) | 0.1742 |
| Antimalarial use at baseline | No | 212 | 0.0 (–24.8, 49.6) | | 244 | 107.0 (44.0, 200.0) | |
| | Yes | 281 | 0.0 (–34.1, 50.0) | 0.3256 | 374 | 126.7 (52.6, 262.5) | 0.0705 |
| Age group, years | 19–45‡ | 308 | 0.0 (–33.3, 42.9) | | 382 | 111.8 (51.9, 220.0) | |
| | ≤18 | 35 | 14.3 (–17.6, 110.9) | 0.0387 | 46 | 216.0 (90.5, 360.0) | 0.0078 |
| | 46–64 | 137 | 7.7 (–25.0, 54.3) | 0.1541 | 184 | 108.6 (41.7, 248.7) | 0.8518 |
| | ≥65 | 13 | 25.1 (–22.2, 37.5) | 0.5276 | 6 | 149.6 (50.0, 238.5) | 0.6892 |
| Race | Other | 307 | 0.0 (–25.5, 40.0) | | 307 | 108.3 (55.6, 217.4) | |
| | Black | 186 | 7.1 (–33.3, 66.7) | 0.6228 | 311 | 135.3 (41.7, 262.5) | 0.3980 |

*SELENA-SLEDAI was used in studies LBSL02, BLISS-76, and PLUTO; SLEDAI-2K was used in EMBRACE.

[†]Defined as “Yes” if at least one low C3/C4 and anti-dsDNA ≥30 IU/mL, and “No” otherwise

[‡]reference group.

Low C4: <16 mg/dL in studies LBSL02 and BLISS-76; <10 mg/dL in EMBRACE and PLUTO.

Low C3: <90 mg/dL in all studies.

Anti-dsDNA, anti-double-stranded deoxyribonucleic acid antibody; C, complement; IQR, interquartile range; MBC, memory B cell; SELENA-SLEDAI, Safety of Estrogens in Lupus National Assessment - SLE Disease Activity Index; SLE, systemic lupus erythematosus; SLEDAI–2K, SLE Disease Activity Index 2000.

**Supplementary Table S2. Migratory processes associated with downregulated DEGs**

| CELL ADHESION *The attachment of a cell, either to another cell or to an underlying substrate such as the extr* | | GeneRatic | BgRatio | pvalue | p.adjust | qvalue | geneID |
|---|---|---|---|---|---|---|---|
| GO:0007159 | leukocyte cell-cell adhesion | 31/388 | 288/1223 | 2,45E-09 | 1,20E-07 | 9,29E-08 | BCL10/ADAM8/TNIP1/PRKAR1A/NFKBIZ/HLA-E/SASH3/DOCK8/PTPN6/HSPD1/CSK/PTPRC/ITGB7/CD47/HSPH1/CORO1A/SYK/CD70/RHOH/IL27RA/HLA-DPB1/HLA-DMB/ITGB1/LGALS3/IRF1/CCR7/HLA-A/RAC2/SELL/NFKBID/CD83 |
| GO:1903039 | positive regulation of leukocyte cell-cell adhesion | 23/388 | 190/1223 | 3,40E-08 | 1,30E-06 | 9,76E-07 | BCL10/ADAM8/NFKBIZ/HLA-E/SASH3/DOCK8/PTPN6/HSPD1/CSK/PTPRC/CD47/HSPH1/CORO1A/SYK/CD70/RHOH/IL27RA/HLA-DPB1/HLA-DMB/CCR7/HLA-A/NFKBID/CD83 |
| GO:1903037 | regulation of leukocyte cell-cell adhesion | 26/388 | 258/1223 | 1,87E-07 | 6,10E-06 | 4,60E-06 | BCL10/ADAM8/PRKAR1A/NFKBIZ/HLA-E/SASH3/DOCK8/PTPN6/HSPD1/CSK/PTPRC/CD47/HSPH1/CORO1A/SYK/CD70/RHOH/IL27RA/HLA-DPB1/HLA-DMB/LGALS3/IRF1/CCR7/HLA-A/NFKBID/CD83 |
| GO:0022409 | positive regulation of cell-cell adhesion | 23/388 | 216/1223 | 3,62E-07 | 1,10E-05 | 8,02E-06 | BCL10/ADAM8/NFKBIZ/HLA-E/SASH3/DOCK8/PTPN6/HSPD1/CSK/PTPRC/CD47/HSPH1/CORO1A/SYK/CD70/RHOH/IL27RA/HLA-DPB1/HLA-DMB/CCR7/HLA-A/NFKBID/CD83 |
| GO:0045785 | positive regulation of cell adhesion | 28/388 | 321/1223 | 1,23E-06 | 3,20E-05 | 2,39E-05 | BCL10/ADAM8/ILK/CIB1/NFKBIZ/HLA-E/SASH3/DOCK8/PTPN6/HSPD1/CSK/CALR/S100A10/PTPRC/CD47/HSPH1/CORO1A/SYK/CD70/ARPC2/RHOH/IL27RA/HLA-DPB1/HLA-DMB/CCR7/HLA-A/NFKBID/CD83 |
| GO:0034109 | homotypic cell-cell adhesion | 11/388 | 63/12238 | 3,95E-06 | 8,60E-05 | 6,49E-05 | MYH9/ILK/PTPN6/ACTG1/ACTB/PPIA/HSPB1/SYK/MYL12A/CLIC1/PLEK |
| GO:0022407 | regulation of cell-cell adhesion | 26/388 | 330/1223 | 1,84E-05 | 0,00033 | 0,000247 | BCL10/ADAM8/PRKAR1A/NFKBIZ/HLA-E/SASH3/DOCK8/PTPN6/HSPD1/CSK/PTPRC/CD47/HSPH1/CORO1A/SYK/CD70/RHOH/IL27RA/HLA-DPB1/HLA-DMB/LGALS3/IRF1/CCR7/HLA-A/NFKBID/CD83 |
| GO:0033627 | cell adhesion mediated by integrin | 7/388 | 51/12238 | 0,00106 | 0,0096 | 0,007231 | CIB1/LPXN/PTPN6/ITGB7/SYK/TESC/ITGB1 |
| GO:1900026 | positive regulation of substrate adhesion-dependent cell spreading | 5/388 | 32/12238 | 0,003103 | 0,022 | 0,01684 | ILK/CIB1/CALR/S100A10/ARPC2 |
| GO:0033628 | regulation of cell adhesion mediated by integrin | 5/388 | 36/12238 | 0,005238 | 0,033 | 0,024749 | CIB1/LPXN/PTPN6/SYK/TESC |
| ACTIN FILAMENT-BASED PROCESS *Any cellular process that depends upon or alters the actin cytoskeleton, that part of the cytoskeleton comprising actin filaments and their associated proteins.* | | | | | | | |
| GO:0032970 | regulation of actin filament-based process | 31/388 | 296/1223 | 4,75E-09 | 2,20E-07 | 1,69E-07 | HCLS1/MYH9/ARHGDIB/CAPZA1/WDR1/ARPC3/CDC42EP3/PFN1/RHOG/ACTG1/ARPC1B/GPR65/S100A10/CD47/ABRACL/TMSB4X/CORO1A/TWF2/GMFG/ARPC2/CAPZB/PDE4D/RHOH/ACTR3/ARPC5/CCR7/BRK1/CAPG/BST2/RAC2/PLEK |
| GO:0032956 | regulation of actin cytoskeleton organization | 28/388 | 271/1223 | 3,64E-08 | 1,40E-06 | 1,02E-06 | HCLS1/ARHGDIB/CAPZA1/WDR1/ARPC3/CDC42EP3/PFN1/RHOG/ACTG1/ARPC1B/GPR65/S100A10/CD47/TMSB4X/CORO1A/TWF2/GMFG/ARPC2/CAPZB/RHOH/ACTR3/ARPC5/CCR7/BRK1/CAPG/BST2/RAC2/PLEK |
| GO:0008064 | regulation of actin polymerization or depolymerization | 19/388 | 149/1223 | 2,37E-07 | 7,40E-06 | 5,61E-06 | HCLS1/CAPZA1/WDR1/ARPC3/CDC42EP3/PFN1/ARPC1B/TMSB4X/CORO1A/TWF2/GMFG/ARPC2/CAPZB/ACTR3/ARPC5/CCR7/BRK1/CAPG/PLEK |
| GO:0030832 | regulation of actin filament length | 19/388 | 150/1223 | 2,64E-07 | 8,10E-06 | 6,13E-06 | HCLS1/CAPZA1/WDR1/ARPC3/CDC42EP3/PFN1/ARPC1B/TMSB4X/CORO1A/TWF2/GMFG/ARPC2/CAPZB/ACTR3/ARPC5/CCR7/BRK1/CAPG/PLEK |
| GO:0110053 | regulation of actin filament organization | 23/388 | 214/1223 | 3,06E-07 | 9,10E-06 | 6,89E-06 | HCLS1/CAPZA1/WDR1/ARPC3/CDC42EP3/PFN1/ACTG1/ARPC1B/GPR65/S100A10/CD47/TMSB4X/CORO1A/TWF2/GMFG/ARPC2/CAPZB/ACTR3/ARPC5/CCR7/BRK1/CAPG/PLEK |
| GO:0008154 | actin polymerization or depolymerization | 20/388 | 175/1223 | 6,89E-07 | 1,90E-05 | 1,45E-05 | HCLS1/CAPZA1/WDR1/WIPF1/ARPC3/CDC42EP3/PFN1/ARPC1B/TMSB4X/CORO1A/TWF2/GMFG/ARPC2/CAPZB/ACTR3/ARPC5/CCR7/BRK1/CAPG/PLEK |
| GO:0051493 | regulation of cytoskeleton organization | 33/388 | 407/1223 | 7,13E-07 | 1,90E-05 | 1,47E-05 | HCLS1/ARHGDIB/CAPZA1/WDR1/CIB1/ARPC3/CDC42EP3/NIN/PFN1/CAPN2/RHOG/ACTG1/ARPC1B/GPR65/S100A10/CD47/TMSB4X/CORO1A/TWF2/GMFG/ARPC2/CHMP5/CAPZB/RHOH/CDK2AP2/ACTR3/ARPC5/CCR7/BRK1/CAPG/BST2/RAC2/PLEK |
| GO:0007015 | actin filament organization | 29/388 | 341/1223 | 1,32E-06 | 3,40E-05 | 2,56E-05 | HCLS1/CAPZA1/WDR1/WIPF1/ARPC3/LCP1/CDC42EP3/PFN1/RHOG/ACTG1/ARPC1B/GPR65/S100A10/CD47/TMSB4X/CORO1A/TWF2/GMFG/ARPC2/CAPZB/MARCKS/RHOH/ACTR3/ARPC5/CCR7/BRK1/CAPG/RAC2/PLEK |
| GO:0030833 | regulation of actin filament polymerization | 17/388 | 136/1223 | 1,36E-06 | 3,40E-05 | 2,61E-05 | HCLS1/CAPZA1/ARPC3/CDC42EP3/PFN1/ARPC1B/TMSB4X/CORO1A/TWF2/GMFG/ARPC2/CAPZB/ACTR3/ARPC5/CCR7/BRK1/CAPG |
| GO:0030041 | actin filament polymerization | 17/388 | 153/1223 | 6,96E-06 | 0,00014 | 0,000109 | HCLS1/CAPZA1/ARPC3/CDC42EP3/PFN1/ARPC1B/TMSB4X/CORO1A/TWF2/GMFG/ARPC2/CAPZB/ACTR3/ARPC5/CCR7/BRK1/CAPG |
| GO:0051495 | positive regulation of cytoskeleton organization | 18/388 | 177/1223 | 1,30E-05 | 0,00024 | 0,000184 | HCLS1/WDR1/ARPC3/CDC42EP3/NIN/PFN1/ARPC1B/GPR65/S100A10/CD47/CORO1A/GMFG/ARPC2/ACTR3/ARPC5/CCR7/BRK1/PLEK |
| GO:0097581 | lamellipodium organization | 11/388 | 73/12238 | 1,72E-05 | 0,00031 | 0,000233 | AKIRIN1/PLEKHO1/TWF2/SNX2/ARPC2/CAPZB/ITGB1/ACTR3/ARPC5/BRK1/RAC2 |
| GO:0030838 | positive regulation of actin filament polymerization | 11/388 | 79/12238 | 3,68E-05 | 0,00062 | 0,00047 | ARPC3/CDC42EP3/PFN1/ARPC1B/CORO1A/GMFG/ARPC2/ACTR3/ARPC5/CCR7/BRK1 |
| GO:0045010 | actin nucleation | 8/388 | 42/12238 | 4,35E-05 | 0,00072 | 0,000544 | ARPC3/ARPC1B/CORO1A/GMFG/ARPC2/ACTR3/ARPC5/BRK1 |
| GO:0010591 | regulation of lamellipodium assembly | 7/388 | 32/12238 | 5,17E-05 | 0,00084 | 0,000635 | AKIRIN1/TWF2/ARPC2/CAPZB/ACTR3/BRK1/RAC2 |
| GO:0034314 | Arp2/3 complex-mediated actin nucleation | 7/388 | 33/12238 | 6,38E-05 | 0,001 | 0,000757 | ARPC3/ARPC1B/GMFG/ARPC2/ACTR3/ARPC5/BRK1 |
| GO:0010592 | positive regulation of lamellipodium assembly | 6/388 | 26/12238 | 0,000131 | 0,0018 | 0,001347 | AKIRIN1/TWF2/ARPC2/ACTR3/BRK1/RAC2 |
| GO:1902743 | regulation of lamellipodium organization | 7/388 | 41/12238 | 0,00027 | 0,0031 | 0,002322 | AKIRIN1/TWF2/ARPC2/CAPZB/ACTR3/BRK1/RAC2 |
| GO:1902745 | positive regulation of lamellipodium organization | 6/388 | 33/12238 | 0,000523 | 0,0054 | 0,004099 | AKIRIN1/TWF2/ARPC2/ACTR3/BRK1/RAC2 |
| GO:0030032 | lamellipodium assembly | 8/388 | 60/12238 | 0,000574 | 0,0059 | 0,004435 | AKIRIN1/TWF2/ARPC2/CAPZB/ITGB1/ACTR3/BRK1/RAC2 |
| GO:0030865 | cortical cytoskeleton organization | 7/388 | 53/12238 | 0,001336 | 0,012 | 0,008738 | WDR1/LCP1/RHOG/CALR/RHOH/RAC2/PLEK |
| GO:0030834 | regulation of actin filament depolymerization | 6/388 | 43/12238 | 0,002207 | 0,017 | 0,013062 | CAPZA1/WDR1/TWF2/CAPZB/CAPG/PLEK |
| GO:0030042 | actin filament depolymerization | 6/388 | 47/12238 | 0,003495 | 0,024 | 0,018233 | CAPZA1/WDR1/TWF2/CAPZB/CAPG/PLEK |
| GO:0051016 | barbed-end actin filament capping | 4/388 | 22/12238 | 0,004625 | 0,03 | 0,022513 | CAPZA1/TWF2/CAPZB/CAPG |
| GO:0030837 | negative regulation of actin filament polymerization | 6/388 | 51/12238 | 0,005274 | 0,033 | 0,024754 | CAPZA1/PFN1/TMSB4X/TWF2/CAPZB/CAPG |
| CELL MIGRATION *The controlled self-propelled movement of a cell from one site to a destination guided by molecular cues. Cell migration is a central process in the development and maintenance of multicellular organisms.* | | | | | | | |
| GO:0050900 | leukocyte migration | 27/388 | 343/1223 | 1,29E-05 | 0,00024 | 0,000184 | ADAM8/MYH9/WDR1/CD48/AKIRIN1/BSG/DOCK8/PTPN6/RHOG/CALR/PPIA/ITGB7/CD47/HMOX1/CORO1A/SYK/GPSM3/JCHAIN/RHOH/IL27RA/ITGB1/PPIB/LGALS3/CCR7/IGKC/RAC2/SELL |
| GO:0097530 | granulocyte migration | 12/388 | 91/12238 | 2,86E-05 | 0,00049 | 0,000373 | ADAM8/WDR1/AKIRIN1/BSG/RHOG/PPIA/SYK/RHOH/PPIB/LGALS3/CCR7/RAC2 |
| GO:1990266 | neutrophil migration | 11/388 | 77/12238 | 2,88E-05 | 0,00049 | 0,000373 | ADAM8/WDR1/BSG/RHOG/PPIA/SYK/RHOH/PPIB/LGALS3/CCR7/RAC2 |
| GO:0030335 | positive regulation of cell migration | 26/388 | 365/1223 | 0,000101 | 0,0015 | 0,001122 | ADAM8/PLCG2/CIB1/FGR/AKIRIN1/DOCK8/PFN1/CALR/ACTG1/GRN/PTPRC/HSPB1/RAB11A/TMSB4X/HMOX1/CORO1A/GPSM3/ATP5F1B/ITGB1/LGALS3/TRADD/CCR7/CD40/S100A11/RAC2/CTSH |
| GO:0001667 | ameboidal-type cell migration | 22/388 | 285/1223 | 0,00011 | 0,0016 | 0,001199 | SP100/ZFAND5/MYH9/ARHGDIB/PLCG2/CIB1/LPXN/PFN1/CALR/GRN/ITGB7/HSPB1/CAP1/RAB11A/TMSB4X/HMOX1/ATP5F1B/ANXA6/ITGB1/NR4A1/CD40/CTSH |
| GO:0010634 | positive regulation of epithelial cell migration | 12/388 | 106/1223 | 0,00013 | 0,0018 | 0,001347 | PLCG2/CIB1/PFN1/CALR/GRN/HSPB1/RAB11A/TMSB4X/HMOX1/ATP5F1B/CD40/CTSH |
| GO:2000147 | positive regulation of cell motility | 26/388 | 378/1223 | 0,000177 | 0,0022 | 0,001674 | ADAM8/PLCG2/CIB1/FGR/AKIRIN1/DOCK8/PFN1/CALR/ACTG1/GRN/PTPRC/HSPB1/RAB11A/TMSB4X/HMOX1/CORO1A/GPSM3/ATP5F1B/ITGB1/LGALS3/TRADD/CCR7/CD40/S100A11/RAC2/CTSH |
| GO:0097529 | myeloid leukocyte migration | 13/388 | 143/1223 | 0,000616 | 0,0062 | 0,00468 | ADAM8/WDR1/AKIRIN1/BSG/RHOG/PPIA/CD47/SYK/RHOH/PPIB/LGALS3/CCR7/RAC2 |
| GO:0010631 | epithelial cell migration | 16/388 | 210/1223 | 0,001072 | 0,0096 | 0,007277 | SP100/MYH9/PLCG2/CIB1/LPXN/PFN1/CALR/GRN/HSPB1/RAB11A/TMSB4X/HMOX1/ATP5F1B/NR4A1/CD40/CTSH |
| GO:0090132 | epithelium migration | 16/388 | 211/1223 | 0,001127 | 0,01 | 0,007615 | SP100/MYH9/PLCG2/CIB1/LPXN/PFN1/CALR/GRN/HSPB1/RAB11A/TMSB4X/HMOX1/ATP5F1B/NR4A1/CD40/CTSH |
| GO:0090130 | tissue migration | 16/388 | 213/1223 | 0,001244 | 0,011 | 0,008231 | SP100/MYH9/PLCG2/CIB1/LPXN/PFN1/CALR/GRN/HSPB1/RAB11A/TMSB4X/HMOX1/ATP5F1B/NR4A1/CD40/CTSH |
| GO:0043536 | positive regulation of blood vessel endothelial cell migration | 6/388 | 39/12238 | 0,001314 | 0,011 | 0,00865 | CIB1/HSPB1/TMSB4X/HMOX1/ATP5F1B/CD40 |
| GO:0010632 | regulation of epithelial cell migration | 13/388 | 162/1223 | 0,001945 | 0,016 | 0,011836 | SP100/PLCG2/CIB1/PFN1/CALR/GRN/HSPB1/RAB11A/TMSB4X/HMOX1/ATP5F1B/CD40/CTSH |
| GO:0010595 | positive regulation of endothelial cell migration | 8/388 | 75/12238 | 0,002508 | 0,019 | 0,014393 | CIB1/CALR/GRN/HSPB1/TMSB4X/HMOX1/ATP5F1B/CD40 |
| GO:0002685 | regulation of leukocyte migration | 12/388 | 149/1223 | 0,002779 | 0,02 | 0,015505 | ADAM8/AKIRIN1/DOCK8/RHOG/CALR/HMOX1/GPSM3/RHOH/IL27RA/LGALS3/CCR7/RAC2 |
| GO:0043542 | endothelial cell migration | 12/388 | 152/1223 | 0,003275 | 0,023 | 0,017306 | SP100/MYH9/CIB1/LPXN/CALR/GRN/HSPB1/TMSB4X/HMOX1/ATP5F1B/NR4A1/CD40 |
| GO:1902622 | regulation of neutrophil migration | 5/388 | 33/12238 | 0,003563 | 0,024 | 0,018382 | ADAM8/RHOG/RHOH/CCR7/RAC2 |
| GO:0043534 | blood vessel endothelial cell migration | 8/388 | 83/12238 | 0,004714 | 0,03 | 0,022771 | MYH9/CIB1/HSPB1/TMSB4X/HMOX1/ATP5F1B/NR4A1/CD40 |
| CELL CHEMOTAXIS *The directed movement of a motile cell guided by a specific chemical concentration gradient. Movement may be towards a higher concentration (positive chemotaxis) or towards a lower concentration (negative chemotaxis).* | | | | | | | |
| GO:0060326 | cell chemotaxis | 17/388 | 193/1223 | 0,000138 | 0,0018 | 0,001389 | ADAM8/AKIRIN1/BSG/BIN2/RHOG/CALR/PPIA/HSPB1/TMSB4X/CORO1A/SYK/GPSM3/PPIB/LGALS3/NR4A1/CCR7/RAC2 |
| GO:0071621 | granulocyte chemotaxis | 8/388 | 75/12238 | 0,002508 | 0,019 | 0,014393 | AKIRIN1/BSG/PPIA/SYK/PPIB/LGALS3/CCR7/RAC2 |
| GO:0030595 | leukocyte chemotaxis | 12/388 | 149/1223 | 0,002779 | 0,02 | 0,015505 | ADAM8/AKIRIN1/BSG/CALR/PPIA/CORO1A/SYK/GPSM3/PPIB/LGALS3/CCR7/RAC2 |
| GO:0030593 | neutrophil chemotaxis | 7/388 | 63/12238 | 0,003661 | 0,025 | 0,018783 | BSG/PPIA/SYK/PPIB/LGALS3/CCR7/RAC2 |

Migratory processes that are significantly associated with the found DEGs categorized by 4 groups: cell adhesion (the attachment of a cell, either to another cell or to an underlying substrate such as the extracellular matrix, via cell adhesion molecules), actin cytoskeleton organization (any cellular process that depends upon or alters the actin cytoskeleton, that part of the cytoskeleton comprising actin filaments and their associated

proteins), cell migration (the controlled self-propelled movement of a cell from one site to a destination guided by molecular cues), or cell chemotaxis (the directed movement of a motile cell guided by a specific chemical concentration gradient).

Arp2/3; Actin Related Protein 2/3; DEG, differentially expressed gene; p.adjust, adjusted p-value.

## Supplementary references


1. ClinicalTrials.gov. Synergetic B-cell immunomodulation in SLE - 2nd study. (SynBioSe-2). 2000. URL: https://clinicaltrials.gov/ct2/show/NCT03747159
2. Teng YKO, Bruce IN, Diamond B, et al. Phase III, multicentre, randomised, double-blind, placebo-controlled, 104-week study of subcutaneous belimumab administered in combination with rituximab in adults with systemic lupus erythematosus (SLE): BLISS-BELIEVE study protocol. BMJ Open 2019;9:e025687.
3. Blanco E, Pérez-Andrés M, Arriba-Méndez S, et al. Age-associated distribution of normal B-cell and plasma cell subsets in peripheral blood. J Allergy Clin Immunol 2018;141:2208–19.e16.
4. Blanco E, Pérez-Andrés M, Arriba-Méndez S, et al. Defects in memory B-cell and plasma cell subsets expressing different immunoglobulin-subclasses in patients with CVID and immunoglobulin subclass deficiencies. J Allergy Clin Immunol 2019;144:809–24.
5. Kalina T, Flores-Montero J, van der Velden VH, et al. EuroFlow standardization of flow cytometer instrument settings and immunophenotyping protocols. Leukemia 2012;26:1986-2010.
6. van Dam LS, Oskam JM, Kamerling SWA, et al. Highly sensitive flow cytometric detection of residual B-cells after rituximab in anti-neutrophil cytoplasmic antibodies-associated vasculitis patients. Front Immunol 2020;11:566732.
7. Ilicic T, Kim JK, Kolodziejczyk AA, et al. Classification of low quality cells from single-cell RNA-seq data. Genome Biol 2016;17:29.

8. Tipton CM, Fucile CF, Darce J, et al. Diversity, cellular origin and autoreactivity of antibody-secreting cell population expansions in acute systemic lupus erythematosus. Nat Immunol 2015;16:755-65.

9. Alamyar E, Duroux P, Lefranc MP, et al. IMGT((R)) tools for the nucleotide analysis of immunoglobulin (IG) and T cell receptor (TR) V-(D)-J repertoires, polymorphisms, and IG mutations: IMGT/V-QUEST and IMGT/HighV-QUEST for NGS. Methods Mol Biol 2012;882:569-604.

10. R Core Team. R: A language and environment for statistical computing. 2017. URL: https://www.R-project.org/

11. Hafemeister C, Satija R. Normalization and variance stabilization of single-cell RNA-seq data using regularized negative binomial regression. Genome Biol. 2019;20(1):296. Published 2019 Dec. doi:10.1186/s13059-019-1874-1.

12. Butler A, Hoffman P, Smibert P, Papalexi E, Satija R. Integrating single-cell transcriptomic data across different conditions, technologies, and species. Nat Biotechnol. 2018;36(5):411-420. doi:10.1038/nbt.4096.

13. Stuart T, Butler A, Hoffman P, et al. Comprehensive Integration of Single-Cell Data. Cell. 2019;177(7):1888-1902.e21. doi:10.1016/j.cell.2019.05.031.

14. Ashburner M, Ball CA, Blake JA, et al. Gene ontology: tool for the unification of biology. The Gene Ontology Consortium. Nat Genet 2000;25:25-9.
